# Inhibition of DYRK1A-EGFR axis by p53-MDM2 cascade mediates the induction of cellular senescence

**DOI:** 10.1038/s41419-019-1521-5

**Published:** 2019-03-25

**Authors:** Xiuhua Xu, Qiao Liu, Chen Zhang, Shuai Ren, Limei Xu, Zixiao Zhao, Hao Dou, Peishan Li, Xiyu Zhang, Yaoqin Gong, Changshun Shao

**Affiliations:** 1 Key Laboratory of Experimental Teratology, Ministry of Education, Department of Molecular Medicine and Genetics, Shandong University School of Medicine, Jinan, Shandong 250012 China; 2 State Key Laboratory of Radiation Medicine and Protection, Institutes for Translational Medicine, Soochow University, Suzhou, Jiangsu 215123 China

## Abstract

Activation of p53 may induce apoptosis or cellular senescence in stressed cells. We here report that epidermal growth factor receptor (EGFR) is downregulated by p53 activation in a subset of cancer cell lines, and this EGFR downregulation mediates cellular senescence caused by p53 activation. EGFR confers resistance to senescence by sustaining the ERK signaling. DYRK1A (dual-specificity tyrosine-phosphorylated and tyrosine-regulated kinase 1A), an EGFR-stabilizing kinase, is downregulated by p53 and, when ectopically expressed, can attenuate p53 activation-induced EGFR reduction and cellular senescence. We further showed that the increased degradation of DYRK1A caused by p53 activation was mediated by MDM2. MDM2 was found to physically interact with and ubiquitinate DYRK1A, ultimately leading to its proteosomal degradation. Importantly, administration of Nutlin-3a, which disrupts the binding of MDM2 to p53, but not that of MDM2 to DYRK1A, reduced the levels of DYRK1A and EGFR, induced senescence, and inhibited growth of tumor xenografts formed by U87 glioblastoma cells. Ectopic expression of EGFR in tumor xenografts attenuated senescence and tumor reduction caused by Nultin-3a. Our findings thus established a novel link between p53 and EGFR and may have implications in p53 activation-based therapies.

## Introduction

Upregulation of epidermal growth factor receptor (EGFR), in the forms of amplification and activating point mutation, was commonly detected in lung cancer^1–3^, gliblastomas^4^, esophageal squamous cell cancers^5^, and many other types of cancer^6^. The gain of function in EGFR plays a critical role in driving the proliferation and survival of many types of cancer cells, via upregulating the AKT and MAPK pathways. Correspondingly, treatment of lung cancers bearing EGFR mutations with EGFR tyrosine kinase inhibitors Gefitinib and Erlotinib has been shown to be much more effective than chemotherapy^7–9^. In addition, upregulation of EGFR in tumor stroma also mediates angiogenesis and resistance to vascular endothelial growth factor (VEGF) inhibitor^10^. Cancer cells can even transfer activated EGFR to macrophages and thereby suppress innate immunity^11^. Therefore, inhibition of EGFR signaling by RTK inhibitor or antibodies has far-reaching clinical implications.

*TP53* is the most commonly mutated tumor suppressor gene in human cancer^12^. p53, the protein encoded by *TP53*, becomes activated in stressed cells and regulates diverse biological processes by functioning as a transcriptional factor. The basal p53 level is low because p53 is rapidly ubiquitinated and degraded by ubiquitin ligase MDM2, which is p53 transcriptional target itself. MDM2 thus negatively regulates p53 in a feedback loop. MDM2 was also found to participate in the ubiquitination of other proteins^13–21^. *EGFR* has been shown to be either up- or downregulated by p53 at the transcription level, depending on cell lines or cell types under study^22–25^. Many factors were also identified to regulate EGFR turnover at protein level^26–28^. Dual-specificity tyrosine-phosphorylated and tyrosine-regulated kinase 1A, or DYRK1A, was shown to promote the stabilization of EGFR by phosphorylating SPRY2, which interferes with the Cbl-mediated ubiquitination of EGFR^29^. Interestingly, DYRK1A can be negatively regulated by p53 via miR-1246^30^. Therefore, diverse mechanisms may govern the regulation of EGFR by p53.

Downregulation of EGFR-MEK-ERK signaling pathway is sufficient to induce cellular senescence in glioblastoma cells^31^. In an effort to elucidate the mechanisms underlying the cellular senescence induced by p53 activation, we found that downregulation of EGFR can also mediate p53-induced senescence in a subset of cancer cell lines. The downregulation of EGFR by p53 is achieved at both the transcriptional level and protein level. Even in cells in which *EGFR* transcription is enhanced by p53 activation, EGFR protein level can still be reduced. DYRK1A, which is required for the maintenance of EGFR stability, is downregulated by p53. We further showed that the downregulation of DYRK1A is mediated by p53 target gene *MDM2*. MDM2 was demonstrated to interact with and polyubiquitinate DYRK1A, leading to its proteosomal degradation. Thus, our results establish a negative regulation of EGFR by p53 via MDM2-mediated downregulation of DYRK1A.

## Results

### Differential regulation of EGFR by p53 in different cancer cell lines

**Fig. 1:**
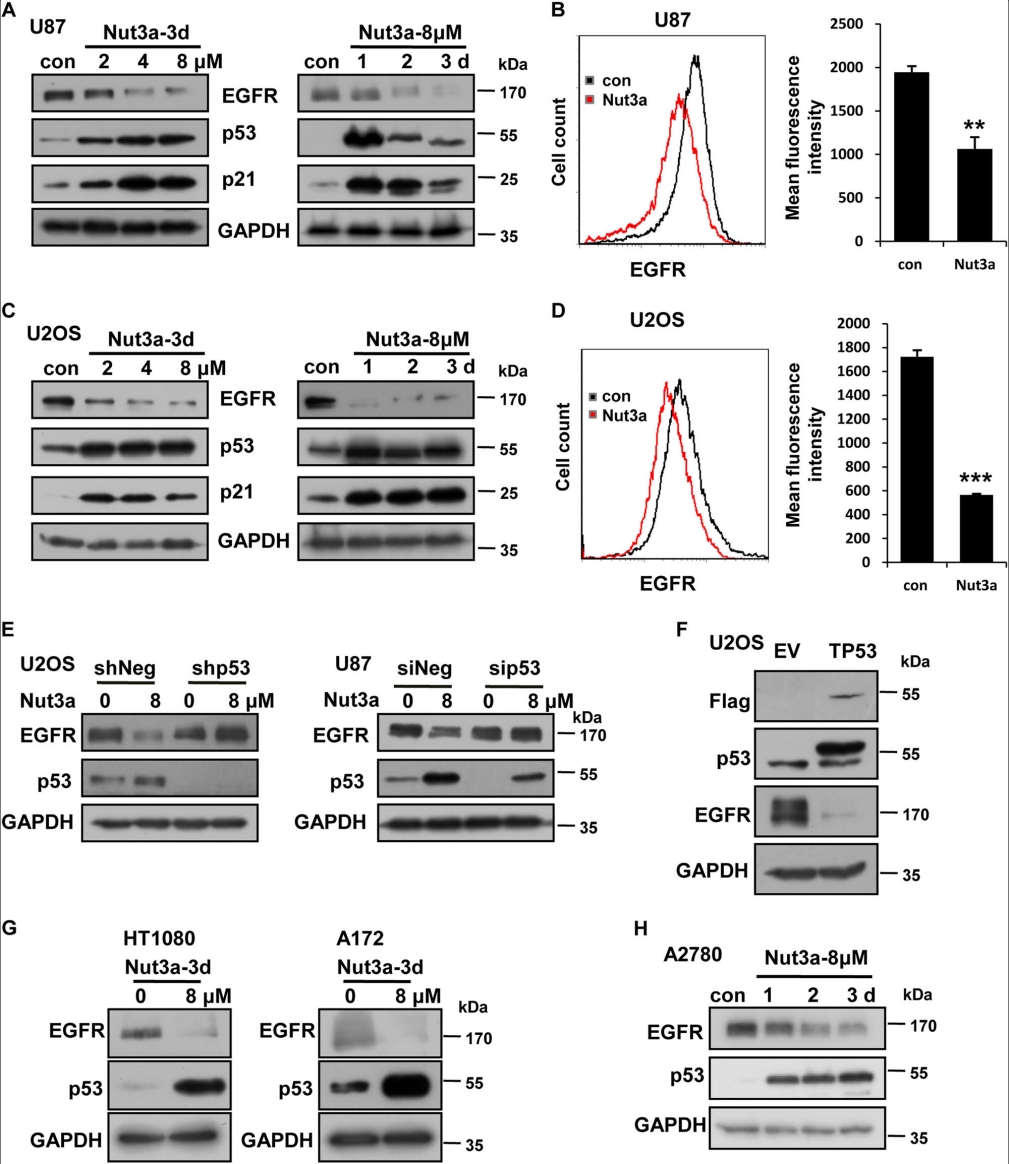
EGFR was downregulated by p53 activation in a subset of cancer cell lines. **a**, **b** Negative regulation of epidermal growth factor receptor (EGFR) by Nutlin-3a (Nut3a) in U87 cells. The protein levels of EGFR were determined by western blot (**a**) or by flow cytometry (**b**, 8 μM for 72 h). Right, quantitative summary of EGFR expression by flow cytometry was shown. Representative results of three independent experiments were shown. **c**, **d** Negative regulation of EGFR by Nut3a in U2OS cells. **c** Western blot analysis of EGFR. **d** Flow cytometry analysis of EGFR in cells treated with Nut3a (8 μM) for 72 h. Right, quantitative summary of EGFR expression by flow cytometry. **e** Reduction of EGFR by Nut3a requires p53 function. Left, the protein levels of EGFR and p53 in U2OS cells transfected with pLKO.1-Puro-shNeg lentiviral vector or pLKO.1-Puro-shp53 lentiviral vector were measured 72 h after treatment with Nut3a. Right, U87 cells were transfected with small interfering RNA (siRNA) duplexes (200 nM) specific to p53 or negative oligo in serum-free medium for 4 h, and then were incubated with complete medium for 48 h. The protein levels of EGFR and p53 in U87 (siNeg and sip53) were measured 72 h after treatment with Nut3a (8 μM). **f** Downregulation of EGFR by ectopic expression of p53. p53 expression vectors were transfected into U2OS cells using Lipofectamine 2000, and cells transfected with empty vectors were as control. After 48 h, the cells extracts were examined by western blot for the determination of Flag, p53, EGFR. **g**, **h** EGFR was downregulated by Nut3a in HT1080, A172, and A2780 cells. The position of protein markers (in kDa) is indicated in each case. Immunoblots are representative of at least two independent experiments with glyceraldehyde 3-phosphate dehydrogenase (GAPDH) serving as a protein loading control. **p < 0.01 vs. control, and ***p < 0.001 vs. control

Both positive and negative regulations of EGFR by p53 have been documented^22–24^. We speculated that the differential regulation by p53 might be cell line-specific. We therefore tested the effect of p53 activation on the level of EGFR by applying Nutlin-3a to cultured cells in a number of commonly used cell lines that carry wild-type TP53. Indeed, different cell lines responded to Nutlin-3a treatment differentially. Consistent with previous reports, treatment by Nutlin-3a led to the upregulation of EGFR in A549 lung cancer cells and MCF-7 breast cancer cells (Fig S1A and S1B). EGFR levels remained unchanged in LoVo and HCT116 colorectal cancer cells (Fig. S1C and S1D). Whereas in U87 glioblastoma cells, p53 activation resulted in a reduction of EGFR, in a time- and dose-dependent manner, as shown by western blot (Fig. 1a). Flow cytometry confirmed the downregulation of EGFR by p53 activation (Fig. 1b). Similarly, EGFR was also downregulated by p53 activation in U2OS osteosarcoma cells (Fig. 1c, d). To confirm that the downregulation of EGFR by Nutlin-3a is p53-dependent, we applied Nutlin-3a to U87 and U2OS cells in which p53 expression was silenced by RNA interference. As shown in Fig. 1e, no reduction in EGFR was detected when p53 was silenced. Furthermore, ectopic expression of p53 also led to a reduction in EGFR (Fig. 1f). In HT1080 fibrosarcoma, A172 gliobastoma, and A2780 ovarian cancer cells, EGFR was also downregulated by Nutlin-3a (Fig. 1g, h). EGFR was also reduced by doxorubicin (Doxo), cisplatin (Cis), or X-rays that activate p53 (Fig. S2A and S2B). However, the level of EGFR appeared to be unaffected by p53 activation in normal human fibroblasts (Fig. S2C). In freshly isolated monocytes and lymphocytes, the protein levels of EGFR were not found to be affected by p53 activation (Fig. S2D-S2F). We suspected that the basal levels of EGFR in monocytes and lymphocytes were already very low so that p53 activation may not be able to further reduce it, and as a positive control, the expression of EGFR protein was detected in U87 cells. Together, these results indicate that EGFR can be downregulated by p53 in many cancer cell lines.

We next determined whether the downregulation of EGFR by p53 occurs at the transcriptional level. In U87 cells, the EGFR messenger RNA (mRNA) was decreased upon p53 activation (Fig. S3A). As a positive control, the mRNA level of *CDKN1A* was increased. A luciferase reporter containing EGFR promoter showed a reduction in luciferase activity when treated by Nutlin-3a (Fig. S3A), indicating that p53 could negatively regulate *EGFR* transcription. However, in contrast to the reduction of EGFR at the protein level, *EGFR* transcription showed a positive response to p53 activation in U2OS and A2780 cells (Fig. S3B and S3C). *EGFR* mRNA levels were reduced by Nutlin-3a in A172 and HT1080 cells (Fig. S3D and S3E). These results suggest that while repression of *EGFR* transcription may contribute to the downregulation of EGFR when p53 is activated, reduction in EGFR can occur in the presence of increased *EGFR* transcription. On the other hand, while the protein amount of EGFR was elevated in A549 cells in response to Nutlin-3a treatment, *EGFR* mRNA level was reduced (Fig. S4). These results suggest that post-transcriptional regulation likely plays an important role in determining the eventual amount of EGFR.

### Downregulation of EGFR mediates cellular senescence induced by p53 activation

**Fig. 2:**
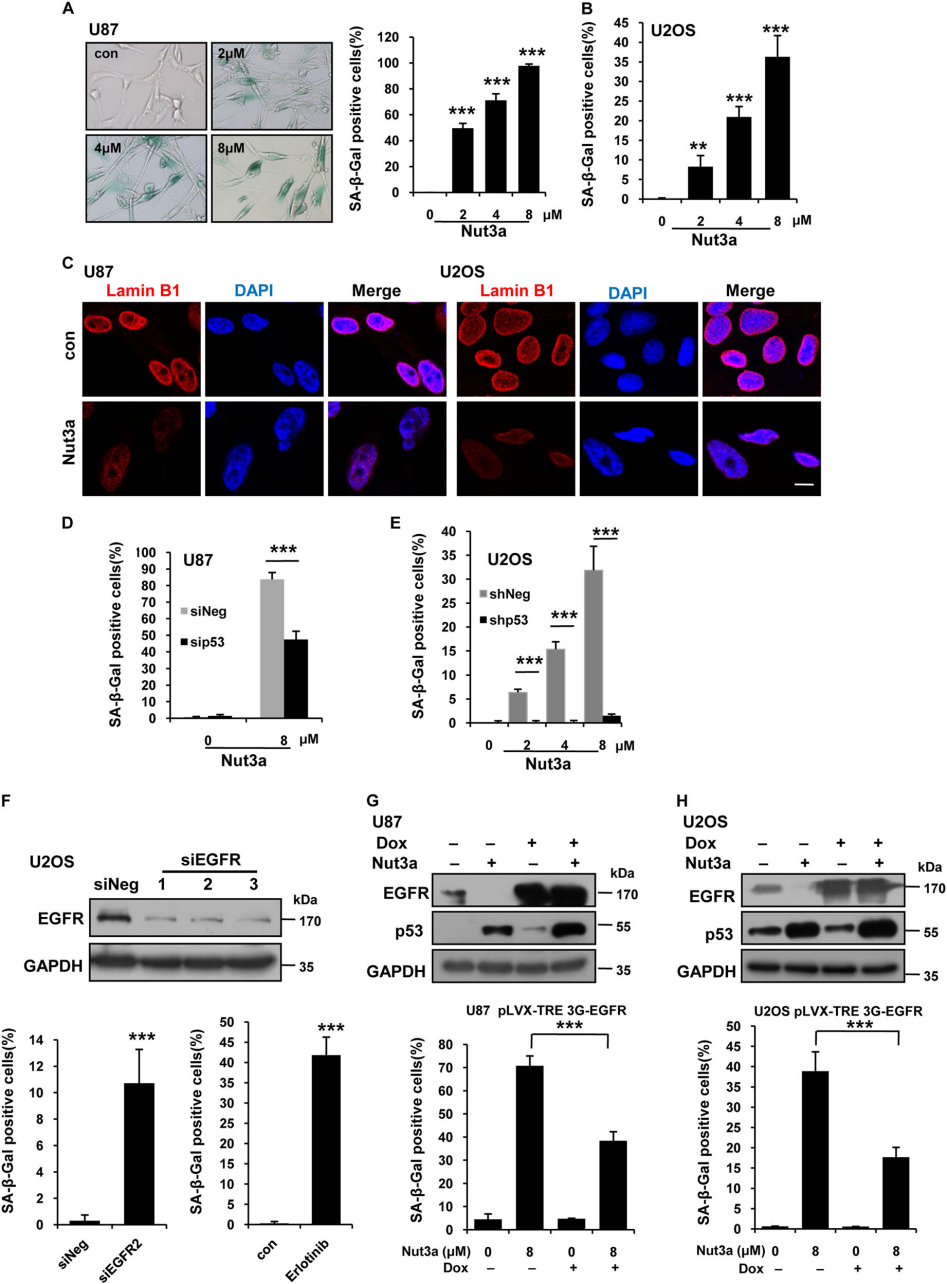
Induction of senescence by p53 activation is mediated by downregulation of EGFR. **a** p53 activation leads to cellular senescence in U87 cells. U87 cells were treated with the indicated concentration of Nut3a for 7 days. Cellular senescence was examined by senescence-associated β-galactosidase (SA-β-gal) staining and quantitative summary of senescent cells was shown. **b** U2OS cells were treated with the indicated concentration of Nut3a for 7 days. Cellular senescence was determined as **a**. **c** Immunofluorescence staining of Lamin B1 in U87 and U2OS treated with 8 μM Nut3a for 7 days. Scale bar, 10 μm. **d** The percentages of SA-β-gal-positive cells were also scored for U87 cells transfected with control or p53 small interfering RNAs (siRNAs) that were treated with 8 μM of Nut3a for 7 days. Data are presented as averages of triplicate measurements. **e** Nut3a-induced senescence was also examined in U2OS cells transfected with pLKO.1-Puro-shNeg or pLKO.1-Puro-shp53 lentiviral vector were treated with Nut3a, showing a p53-dependent effect of Nut3a. **f** Epidermal growth factor receptor (EGFR) siRNA or EGFR inhibitor (Erlotinib) induces senescence in U2OS cells. Top, downregulation of EGFR by siRNA were measured by western blot. Bottom, quantitative summary of senescent cells was shown. Cellular senescence was examined 7 days after EGFR RNA interference (RNAi) or after cells were treated with EGFR inhibitor Erlotinib (10 μM) for 7 days. **g**, **h** Overexpression of EGFR attenuates Nut3a-induced senescence. pLVX-TET 3G and pLVX-TRE 3G-EGFR-inducible expression vectors were transfected into U87 and U2OS cells. Stably transfected cells were obtained by selection with puromycin. Cells were treated with doxycycline (Dox) (200 ng/mL) for 12 h to induce the expression of EGFR. The cells without Dox treatment were used as control. The inducible expression of EGFR was confirmed by western blot. EGFR expressing and control U87 cells were treated with 8 μM Nut3a for 72 h; the corresponding U2OS cells were with 8 μM Nut3a for 7 days before they were processed for SA-β-gal staining. For the SA-β-gal data: means ± SD are representative of three independent experiments. **p < 0.01 vs. control, and ***p < 0.001 vs. control

The activation of p53 can either lead to apoptosis or cellular senescence depending on cell types. We next examined the fates of the cells in which EGFR was downregulated by p53 activation. Nutlin-3a treatment strikingly induced cellular senescence in U87 and U2OS cells, as shown by positive senescence-associated β-galactosidase (SA-β-gal) staining, reduction of lamin B1, and reduced 5-ethynyl-2′-deoxyuridine (EdU) incorporation, p16 (Fig. 2a–c, Figs. S5A–5D). Consistently, depletion of p53 by RNA interference (RNAi) greatly attenuated the Nutlin-3a-induced senescence (Fig. 2d, e). No increase in the level of apoptosis was detected (data not shown). The senescent cells appeared to be arrested at the G0 or G2 phase (data not shown). We previously showed that berberine-induced senescence of U87 glioblastoma cells is mediated by the downregulation of EGFR and RNAi of EGFR alone can induce senescence^31^. We observed that RNAi of EGFR or EGFR inhibitor similarly induced senescence in U2OS (Fig. 2f), HT1080 (Fig S6A), and A172 cells (Fig S6B). To further confirm that downregulation of EGFR mediates p53-induced senescence, we established cell lines that express EGFR upon induction by doxycycline. Indeed, ectopic expression of EGFR in U87 or U2OS cells significantly attenuated senescence induced by p53 activation (Fig. 2g, h). Together, these results indicate that downregulation of EGFR can mediate senescence induced by p53 activation.

We also examined the fates of A549 and MCF-7 cells in which EGFR was upregulated by p53 activation. Interestingly, those cells also predominantly underwent cellular senescence in response to Nutlin-3a (Fig. S6C, S6D). These results indicate that while EGFR downregulation suffices as a cause of cellular senescence and can act downstream of p53 to drive senescence, senescence can occur independent of EGFR. It should be noted that LoVo and HCT116 colorectal cancer cells, in which EGFR remained unchanged in response to p53 activation, exhibited a remarkable increase in apoptotic cell death (data not shown). These results indicate that the cellular senescence-apoptosis cell fate decision is differentially regulated in different cell lines.

### EGFR-stabilizing DYRK1A is negatively regulated by p53

**Fig. 3:**
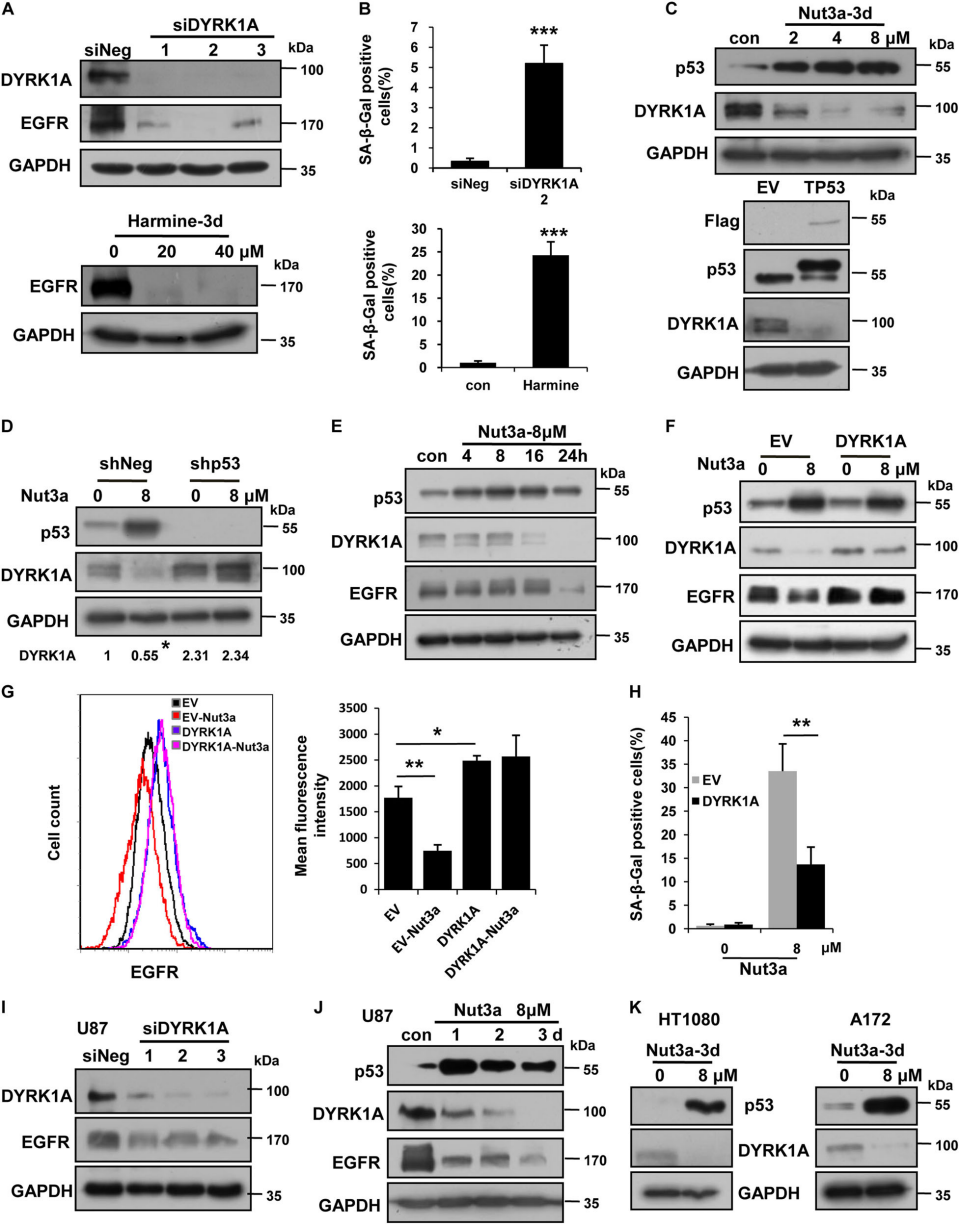
EGFR-stabilizing DYRK1A is negatively regulated by p53. **a** Epidermal growth factor receptor (EGFR) is downregulated by the depletion or inhibition of dual-specificity tyrosine-phosphorylated and tyrosine-regulated kinase 1A (DYRK1A) in U2OS cells. Top, EGFR is downregulated by the depletion of DYRK1A in U2OS cells, as measured by western blot. Bottom, U2OS cells were treated with the indicated concentration of harmine (DYRK1A inhibitor) for 72 h. **b** DYRK1A small interfering RNA (siRNA) and harmine, respectively, induced senescence in U2OS cells. Cellular senescence was examined 7 days after RNA interference (RNAi) of DYRK1A or harmine (20 μM) treatment in U2OS cells. **c** p53 negatively regulates DYRK1A. Top, the protein levels of DYRK1A and p53 in U2OS cells treated with the indicated concentration of Nut3a for 72 h. Bottom, ectopic of p53 expression downregulates DYRK1A. At 48 h after transfection, cell extracts were examined by western blot for the determination of Flag, p53, and DYRK1A. **d** Nut3a failed to downregulate DYRK1A in p53-knockdown cells. The protein levels of DYRK1A and p53 in U2OS cells transfected with pLKO.1-Puro-shNeg lentiviral vector or pLKO.1-Puro-shp53 lentiviral vector were measured 72 h after treatment with Nut3a. **e** Kinetics of DYRK1A and EGFR downregulation by p53 activation. The protein levels of DYRK1A, EGFR and p53 in U2OS cells were determined at different time points after Nut3a treatment. **f** Ectopic expression of DYRK1A attenuates Nut3a-induced downregulation of EGFR, as measured by western blot. pEZ-M77-DYRK1A expression vector was transfected into U2OS cells using Lipofectamine 2000, and cells transfected with pEZ-M77-EV (empty vector) were as control. Cells with stable ectopic expression of DYRK1A were obtained after G418 selection. Cell extracts obtained 72 h after Nut3a treatment was subjected to western blot. **g** Ectopic expression of DYRK1A attenuates Nut3a-induced downregulation of EGFR, as measured by flow cytometry. Cells were treated as in **f**. Representative fluorescence intensity curves and histograms were shown. **h** Ectopic expression of DYRK1A attenuates Nut3a-induced cellular senescence. Cellular senescence was examined 7 days after Nut3a treatment. **i** EGFR is downregulated by the depletion of DYRK1A in U87 cells. **j** p53 activation negatively regulates DYRK1A and EGFR in U87 cells. **k** p53 activation negatively regulates DYRK1A in HT1080 and A172 cells. * *p* < 0.05, **p < 0.01, ***p < 0.001 vs. control

The observation that EGFR protein amount was diminished by p53 activation in some cancer cells, while *EGFR* transcription was elevated suggests that p53 may reduce the stability of EGFR. It was reported that DYRK1A was critical in maintaining the stability of EGFR^27^. We therefore tested whether DYRK1A was involved in the negative regulation of EGFR by p53. Indeed, depletion of DYRK1A by RNAi as well as its inhibition by harmine resulted in a significant reduction in EGFR (Fig. 3a). Consequently, the percentage of senescent cells was significantly increased by either treatment (Fig. 3b). Importantly, DYRK1A was negatively regulated by p53. As shown in Fig. 3c, activation of p53 by Nutlin-3a led to a significant reduction in DYRK1A. Ectopic expression of p53 similarly decreased the level of DYRK1A. As expected, the reduction in DYRK1A caused by Nutlin-3a treatment was greatly attenuated in p53-knockdown cells (Fig. 3d). Furthermore, while DYRK1A and EGFR were both reduced by Nutlin-3a, they occurred sequentially, with the reduction of DYRK1A preceding that of EGFR (Fig. 3e), in agreement with the interpretation that EGFR change is consequential to the downregulation of DYRK1A. As expected, ectopic expression of DYRK1A blocked the reduction in EGFR caused by p53 activation (Fig. 3f, g). Furthermore, DYRK1A greatly rescued p53 activation-induced cellular senescence (Fig. 3h), substantiating the notion that inhibition of DYRK1A-EGFR axis mediates the induction of senescence caused by p53 activation.

We next tested whether DYRK1A would also promote EGFR stability in U87 cells, in which *EGFR* transcription is repressed by p53. As shown in Fig. 3i, knockdown of DYRK1A similarly resulted in a decrease in the amount of EGFR. DYRK1A was also significantly downregulated by p53 activation (Fig. 3j). These results suggest that the downregulation of DYRK1A may still mediate the reduction of EGFR even when *EGFR* transcription is already directly repressed by p53. Furthermore, DYRK1A was also downregulated by p53 activation in HT1080 and A172 cells (Fig. 3k). Therefore, the downregulation of DYRK1A may serve as a common mechanism underlying the reduction of EGFR by p53 activation.

### Downregulation of EGFR-ERK signaling pathway mediates the induction of senescence by p53 activation

**Fig. 4:**
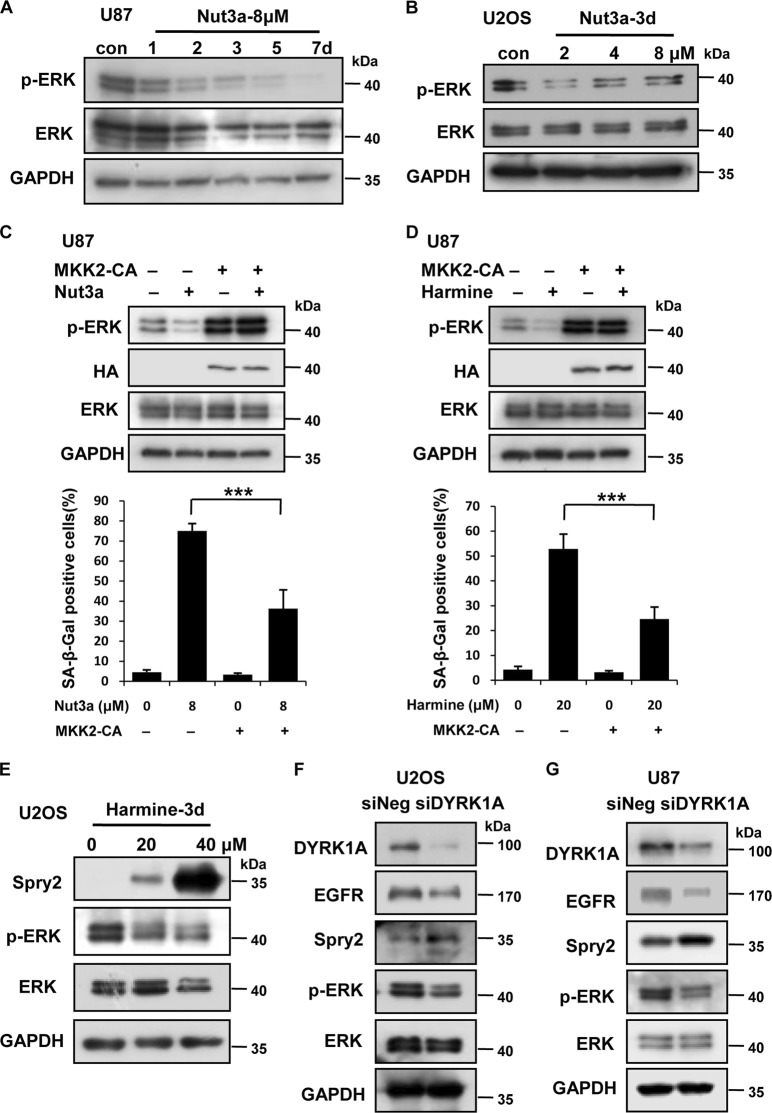
Downregulation of the EGFR-ERK signaling pathway mediates the induction of senescence by p53 activation. **a**, **b** The protein levels of phosaphorylated (p)-ERK and ERK in U87 and U2OS cells were measured by western blot. U87 cells were treated with the indicated time of 8 μM Nut3a, and U2OS cells were treated with the indicated concentration of Nut3a for 3 days. **c** Overexpression of MKK2 attenuates Nut3a-induced senescence. MKK2-CA expression vectors were transfected into U87 cells using Lipofectamine 2000. After expression for 48 h, add media with hygromycin B to get a good stably transfected cells. Then, the cells were treated with 8 μM Nut3a for 72 h. Cell extracts were examined by western blot for the determination of p-ERK, ERK, and HA-MKK2-CA protein levels. Bottom, stable MKK2-CA expression cells were treated with 8 μM Nut3a for 3 days. Cell senescence was examined by senescence-associated β-galactosidase (SA-β-gal) activity analysis. **d** The MKK2-CA expression cells were treated with 20 μM harmine for 72 h. Cell extracts were examined by western blot for the determination of p-ERK, ERK, and HA-MKK2-CA protein levels. Bottom, stable MKK2-CA expression cells were treated with 20 μM harmine for 3 days. Cell senescence was examined by SA-β-gal activity analysis. **e**–**g** p-ERK is downregulated by the depletion or inhibition of dual-specificity tyrosine-phosphorylated and tyrosine-regulated kinase 1A (DYRK1A) in U2OS or U87 cells. **c** U2OS cells were treated with the indicated concentration of harmine for 72 h. p-ERK, ERK, and Spry2 were measured by western blot. **d**, **e**, U2OS and U87 cells were transfected with small interfering RNA (siRNA) specific to DYRK1A or negative oligo for 48 h. The protein levels of DYRK1A, epidermal growth factor receptor (EGFR), Spry2, p-ERK, and ERK were measured by western blot. *** *p* < 0.001  vs. control

We previously showed that the downregulation of the EGFR-MEK-ERK signaling pathway mediated the induction of senescence in berberine-treated glioma cells^31^. We therefore tested whether EGFR-MEK-ERK signaling pathway was also downregulated by p53 activation and mediated the induction of senescence. Indeed, the level of phosphorylated (active) form of ERK was decreased in Nutlin-3a treated U87 and U2OS cells (Fig. 4a, b). To confirm that the downregulation of ERK mediated p53-induced senescence, we established U87 cells that stably express constitutive active MKK2 (MKK2-CA). As shown in Fig. 4c, d, inhibition of ERK activation by Nutlin-3a or harmine was remarkably relieved in the presence of MKK2-CA. Consequently, Nutlin-3a-induced senescence was greatly attenuated in MKK2-CA cells (Fig. 4c). The expression of MKK2-CA in U87 cells also significantly attenuated senescence induced by harmine (Fig. 4d). It was reported that DYRK1A promoted EGFR stabilization through the phosphorylation of Spry2^32^. Spry is tyrosine phosphorylated and competes with EGFR for binding to c-Cbl, an E3-ubiquitin ligase. By interacting with c-Cbl, Spry becomes ubiquitinylated and eventually degraded, allowing for sustained EGFR signaling^33^. Indeed, we observed that inhibition of DYRK1A increased Spry2 and decreased the levels of EGFR and phosphorylated (p)-ERK in U2OS cells (Fig. 4e). Depletion of DYRK1A similarly dampened the EGFR-ERK axis in U2OS and U87 cells (Fig. 4f, g). Together, these results indicate that downregulation of EGFR-ERK signaling pathway mediates the induction of senescence by p53 activation.

### Negative regulation of DYRK1A by p53 is mediated by MDM2

**Fig. 5:**
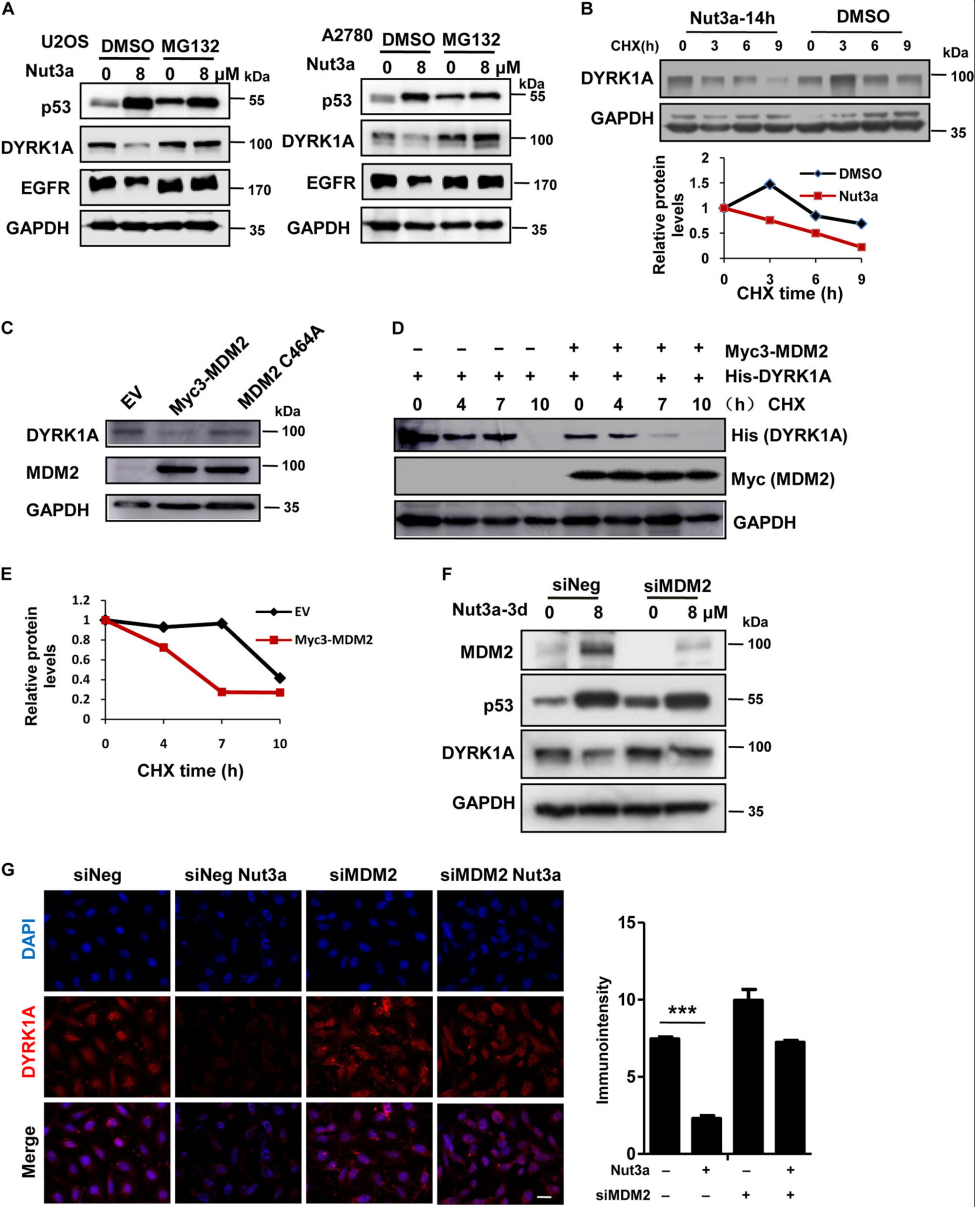
Negative regulation of DYRK1A by p53 is mediated by MDM2. **a** Downregulation of dual-specificity tyrosine-phosphorylated and tyrosine-regulated kinase 1A (DYRK1A) by p53 activation is attenuated by MG132. U2OS and A2780 cells were treated with 8 μM Nut3a for 48 h, and were then treated with 10 μM MG132 for the last 6 h before harvest. Protein levels of p53, epidermal growth factor receptor (EGFR), and DYRK1A were analyzed by western blot. **b** Nut3a shortens the half-life of DYRK1A proteins. U2OS cells were treated with dimethyl sulfoxide (DMSO) or 8 μM Nut3a for 17 h, and were then treated with 50 μg/mL cycloheximide for the indicated durations before harvest. Protein levels of DYRK1A were analyzed by western blot. Bottom: Signals on the immunoblots were analyzed by the ImageJ software (NIH, Bethesda, MD, USA)^45^ and the DYRK1A protein amounts were normalized with that of glyceraldehyde 3-phosphate dehydrogenase (GAPDH). **c** Ectopic expression of MDM2 downregulates DYRK1A. pCMV-myc3-HDM2 (WT) and MDM2 C464A expression vectors were respectively transfected into U2OS cells using Lipofectamine 2000, and cells transfected with empty vector were as control. After 48 h, the cells extracts were examined by western blot for the determination of MDM2, DYRK1A. **d** MDM2 shortens the half-life of DYRK1A proteins. Co-transfection of His-tagged DYRK1A and Myc-tagged MDM2 or empty vector into U2OS cells, and cells were then treated with 50 μg/mL cycloheximide for the indicated durations before harvest. Protein levels of DYRK1A and MDM2 were analyzed by western blot. **e** Signals on the immunoblots of Fig. 5d were analyzed by the ImageJ software (NIH, Bethesda, MD, USA)^44^ and the DYRK1A protein amounts were normalized with that of GAPDH. **f** Downregulation of DYRK1A by p53 activation requires MDM2. U2OS cells were transfected with small interfering RNA (siRNA) duplexes (200 nM) specific to MDM2 or negative oligo in serum-free medium for 4 h, and then were incubated with complete medium for 48 h. The protein levels of MDM2, p53, and DYRK1A in U2OS (siNeg and siMDM2) were measured 72 h after treatment with Nut3a. **g** Downregulation of DYRK1A by p53 activation requires MDM2, as determined by immunofluorescence. Scale bar, 10 μm. Right, quantitative immunointensity of DYRK1A was analyzed by the ImageJ software (NIH, Bethesda, MD, USA) *** *p* < 0.001  vs. control

The downregulation of DYRK1A by p53 activation was not due to decreased transcription (Fig. S7A). It was previously reported that p53 can downregulate DYRK1A by transactivating miR-1246^30^. We therefore tested whether the downregulation of DYRK1A was mediated by miR-1246. Although miR-1246 mimics could indeed lead to a reduction of DYRK1A (Fig. S7B), we detected no increase in miR-1246 expression in U2OS cells treated with Nutlin-3a, though, as a positive control, increased expression was detected in A2780 cells that were used in the previous report (Fig S7C). We next explored whether the reduction of DYRK1A was due to increased proteosomal degradation. MG132 could indeed attenuate the downregulation of DYRK1A caused by p53 activation (Fig. 5a). Corresponding to the block in DYRK1A degradation, EGFR level was not reduced by Nutlin-3a in the presence MG132 (Fig. 5a). It was previously reported that EGFR was subjected to lysosomal degradation^34,35^. We also showed that chloroquine treatment, which blocks lysosomal function, could indeed prevent EGFR degradation in response to Nutlin-3a (Fig. S8A–S8C). Furthermore, examination of protein half-life in the presence of cycloheximide (CHX) showed that the degradation of DYRK1A was greatly accelerated by Nutlin-3a (Fig. 5b). Because MDM2, a p53 transcriptional target, is an ubiquitin ligase, we next tested whether MDM2 plays any role in the degradation of DYRK1A. Strikingly, ectopic expression of MDM2, but not that of mutant MDM2 (C464A), greatly reduced DYRK1A (Fig. 5c, Fig. S8D). Importantly, co-transfection of MDM2 and DYRK1A led to increased degradation of DYRK1a (Fig. 5d, e). Furthermore, DYRK1A was downregulated in U87 and A172 cells when MDM2 was upregulated by Nutlin-3a (Fig. S8E). Depletion of MDM2 by RNAi, on the other hand, blocked the effect of Nutlin-3a (Fig. 5f, g). These results suggest that the negative regulation of DYRK1A by p53 is mediated by MDM2.

### MDM2 interacts with and ubiquitinates DYRK1A

**Fig. 6:**
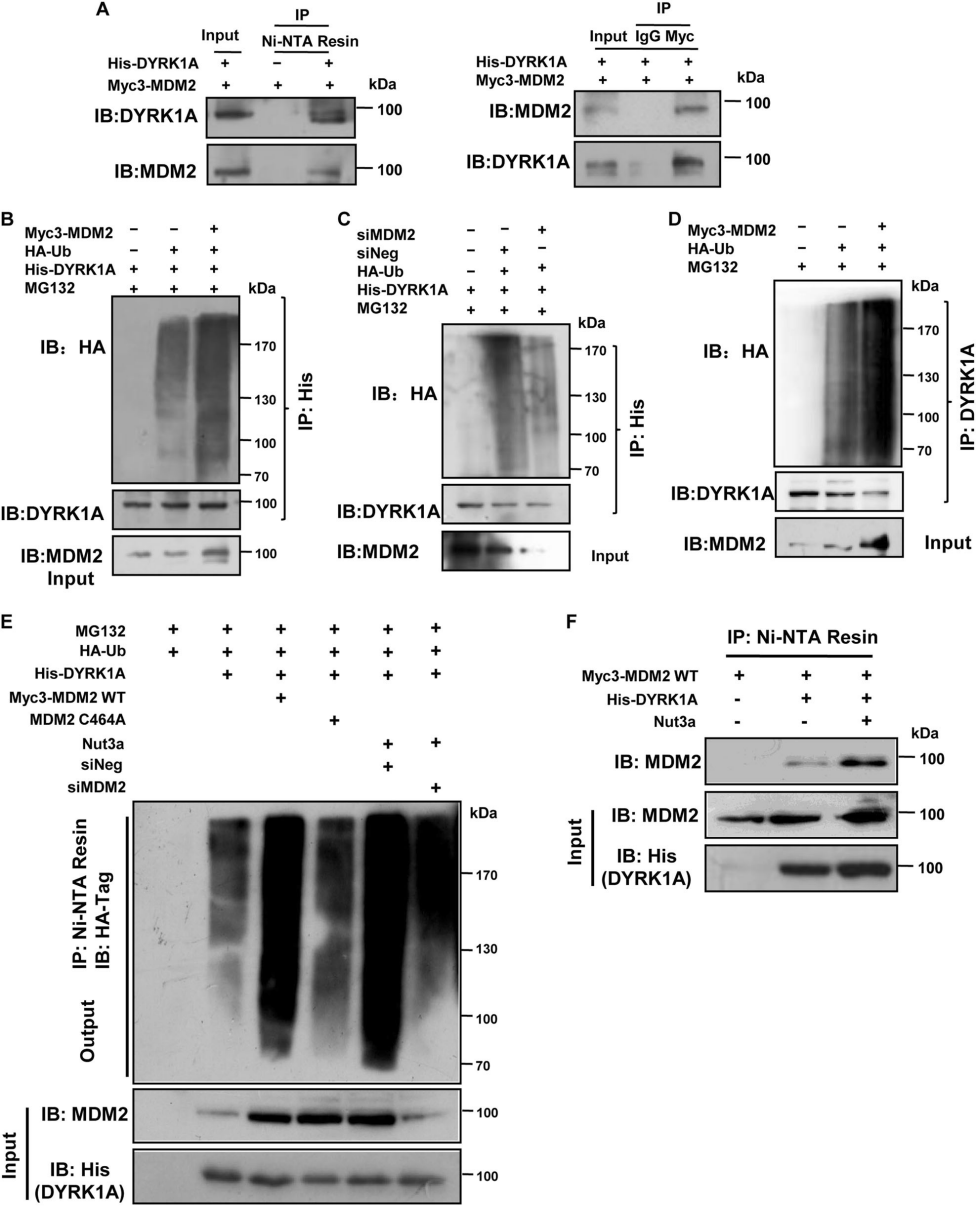
MDM2 interacts with DYRK1A and catalyzes ubiquitination of DYRK1A. **a** MDM2 physically interacts with dual-specificity tyrosine-phosphorylated and tyrosine-regulated kinase 1A (DYRK1A). Left, DYRK1A interacts with MDM2, HEK293T cells were transfected with MDM2 plasmids and the indicated His-DYRK1A or empty vector, and subjected to immunoprecipitation with Ni-NTA, the resulting precipitates as well as a portion (10% of the input) of the cell lysates were immunoblotted with the indicated antibodies. Right, MDM2 interacts with DYRK1A, HEK293T cells were transfected with MDM2 and His-DYRK1A plasmids, and subjected to immunoprecipitation with anti-Myc antibody, the resulting precipitates as well as a portion (10% of the input) of the cell lysates were immunoblotted with the indicated antibodies. **b** MDM2 catalyzes DYRK1A ubiquitination. U2OS cells were transfected with HA-Ub, His-DYRK1A, and empty vector or MDM2 plasmids in the presence of MG132 for 6 h, and cell lysates were then immunoprecipitated with Ni-NTA followed by immunoblotting with an anti-ubiquitin antibody. **c** U2OS cells were transfected with HA-Ub, His-DYRK1A, and siNeg or small interfering RNA (siRNA) of MDM2 in the presence of MG132 for 6 h, and cell lysates were then immunoprecipitated with Ni-NTA followed by immunoblotting with an anti-ubiquitin antibody. **d** MDM2 catalyzes endogenous DYRK1A ubiquitination. U2OS cells were transfected with HA-Ub, and empty vector or MDM2 plasmids in the presence of MG132 for 6 h, and cell lysates were then immunoprecipitated with DYRK1A antibody followed by immunoblotting with an anti-ubiquitin antibody. **e** Wild-type, but not mutant, MDM2 E3 ligase increases the ubiquitination of DYRK1A. Transfection of wild-type MDM2 to U2OS cells enhanced the ubiquitination of DYRK1A (third lane). Transfection of MDM2 C464A that lacked the RING domain for E3 ligase activity failed to do so (fourth lane). His-DYRK1A and HA-Ub with either Myc3-MDM2 or MDM2 C464A were transfected and maintained for 48 h. Cells were treated with MG132 for 6 h before harvesting. Depletion of MDM2 attenuated the degradation of DYRK1A induced by Nut3a. His-DYRK1A and HA-Ub with either siNeg (fifth lane) or siMDM2 (sixth lane) were transfected and maintained for 48 h followed by Nut3a treatment. The cell lysates were immunoprecipitated with Ni-NTA and immunoblotted with anti-HA antibody. **f** DYRK1A interaction with MDM2 was not impaired by Nut3a. U2OS cells were transfected with His-DYRK1A and Myc3-MDM2 plasmids followed by Nut3a treatment, and cell lysates were then immunoprecipitated with Ni-NTA, the resulting precipitates as well as a portion (10% of the input) of the cell lysates were immunoblotted with the indicated antibodies

We next determined whether MDM2 physically interacts with DYRK1A by transfecting HEK293T cells with His-tagged DYRK1A and Myc-tagged MDM2 expression vectors. As shown in Fig. 6a, the two proteins could indeed be reciprocally co-immunoprecipitated. We next determined whether MDM2 could polyubiquitinate DYRK1A. As a positive control, the polyubiquitination of p53 was significantly increased by the ectopic expression of MDM2 (Fig. S9). Similarly, the amount of polyubiquitinated DYRK1A was increased by MDM2 (Fig. 6b). Depletion of MDM2, on the other hand, led to decreased ubiquitination of DYRK1A (Fig. 6c). Ubiquitination of the endogenous DYRK1A was also found to be augmented by the ectopically expressed MDM2 (Fig. 6d). We further tested the ability of MDM2 to polyubiquitinate DYRK1A in U2OS cells. As shown in Fig. 6e, ectopic expression of wild-type MDM2, but not that of mutant MDM2 (C464A), significantly increased the amount of polyubiquitinated DYRK1A. Moreover, the ubiquitination of DYRK1A was greatly increased by Nutlin-3a. However, when MDM2 was depleted, the DYRK1A ubiquitination was also reduced, further supporting that the increased ubiquitination of DYRK1A caused by p53 activation was dependent on MDM2.

Nutlin-3a interrupts MDM2-p53 binding by occupying the p53-binding pocket in MDM2^24^. Therefore, Nutlin-3a is expected to only disrupt the MDM2-p53 binding, but not the MDM2-DYRK1A binding. Indeed, co-immunoprecipitation assay indicates an intact MDM2-DYRK1A binding in the presence of Nutlin-3a (Fig. 6f). Together, these results indicate that p53 activation by Nutlin-3a led to the increased expression of MDM2, and consequently the increased ubiquitination of DYRK1A.

### Nutlin-3a reduces EGFR and DYRK1A in glioblastoma in vivo

**Fig. 7:**
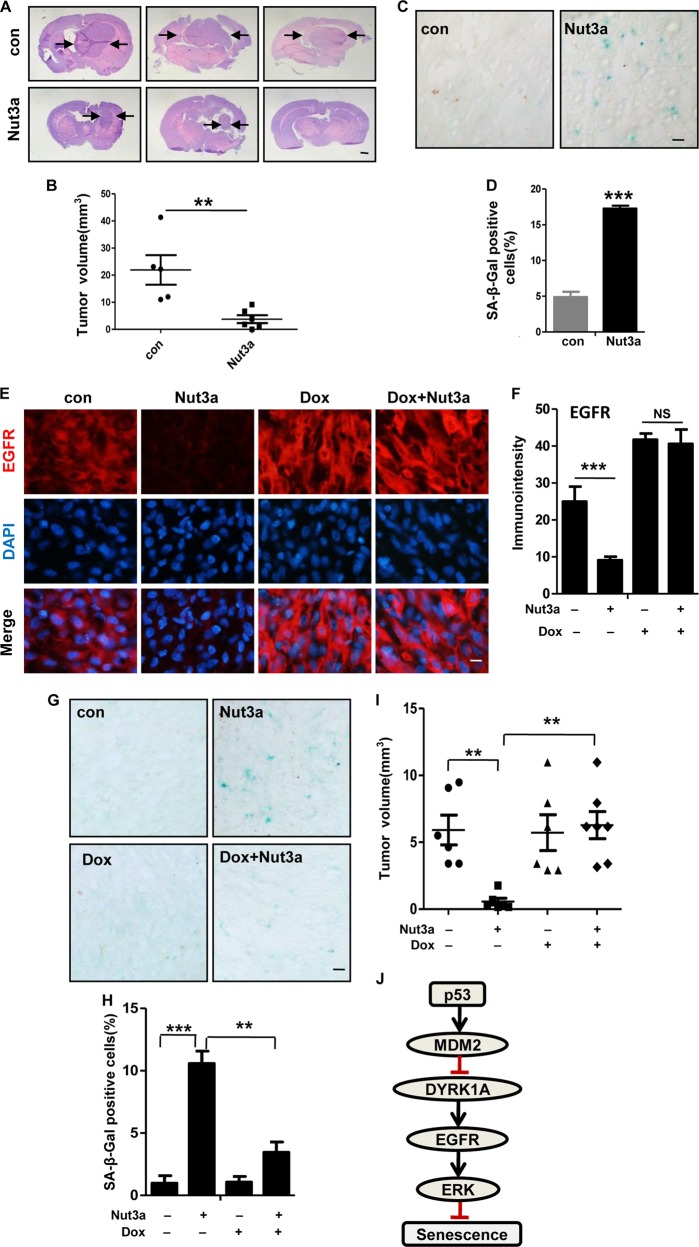
Induction of senescence by p53 activation is mediated by EGFR in vivo. **a**–**d** U87 cells were inoculated into the right striatum of mouse brain (*n* = 5–6 per group). At day 5, 40 mg/kg Nut3a were intraperitoneally injected into mice every other day, on day 28, all mice were euthanized. Brains were fixed, frozen, and systematically sectioned throughout the tumor injection site. Frozen sections (7 μm) were obtained and immunochemical staining was performed. **a** Representative pictures of hematoxylin–eosin staining of tumor sections from mice of two groups (×10). Arrows show the edge of tumor. Scale bar, 1 mm. **b** Tumor volumes were calculated and Nut3a-administered mice exhibited decreased tumor volumes. **c** Representative images showed the positive senescence-associated β-galactosidase (SA-β-gal) staining of tumor sections from Nut3a-administered mice (×400). Scale bar, 20 μm. **d** Quantitative summary of senescent cells was analyzed by the ImageJ software. **e**–**i** U87 pLVX-TRE 3G-EGFR (epidermal growth factor receptor) cells were inoculated into the right striatum of mouse brain (*n* = 6–7 per group).To induce the expression of EGFR, 1.2 mg/mL of doxycycline administered in 5% sucrose-containing drinking water can be used every day. At day 5, 40 mg/kg Nut3a were intraperitoneally injected into mice every other day, and on day 26, all mice were euthanized. **e** Representative immunofluorescence (IF) images showing the expression of EGFR (×400). Scale bar, 10 μm. **f** Quantitative immunointensity of EGFR was analyzed by the ImageJ software. **g**, **h** Overexpression of EGFR attenuates Nut3a-induced senescence. **g** Representative images showed the positive SA-β-gal staining of tumor sections. Scale bar, 20 μm. **h** Quantitative summary of senescent cells was analyzed by the ImageJ software. **i** Tumor volumes were calculated for hematoxylin–eosin staining of tumor sections from mice of four groups. **j** Schematic model of signaling pathways in Nut3a-induced cell senescence. **p < 0.01, ***p < 0.001 vs. control. NS not significant

We next tested whether p53 activation would reduce tumor growth in vivo and whether it would also lead to a downregulation of DYRK1A-EGFR signaling cascade. To this end, we inoculated U87 glioblastoma cells into the right striatum of mouse brains (*n* = 5– 6 per group). On day 5 after tumor cell inoculation, we administered Nutlin-3a at 40 mg/kg by intraperitoneal (i.p.) injection every other day for 23 days. As shown in Fig. 7a, b, tumor size was greatly reduced in the treatment group when compared to control. No tumor was observed in one of the six mice in the treatment group. Significant increase in senescence was detected in the tumors of the treatment group (Fig. 7c, d). As an indicator of p53 activation by Nutlin-3a, p21 was detected in the treatment group (Fig. S10A). Importantly, both DYRK1A and EGFR were reduced drastically in the tumors in the treatment group, as shown by immunofluorescence staining (Fig. S10B and S10C). These results indicate that p53 activation is able to downregulate DYRK1A and EGFR in vivo.

To confirm that downregulation of EGFR mediates p53-induced senescence in vivo, we inoculated U87 glioblastoma cells that express doxycycline-inducible EGFR (U87 pLVX-TRE 3G-EGFR) into the right striatum of mouse brains (*n* = 5– 7 per group). EGFR expression was induced by 1.2 mg/mL of doxycycline administered in 5% sucrose-containing drinking water. On day 5 after tumor cell inoculation Nutlin-3a at 40 mg/kg was administered by i.p. injection every other day for 21 days. As shown in Fig. 7e, f, the ectopically expressed EGFR in the tumor grafts was refractory to Nutlin-3a treatment and remained at a high level. Importantly, the ectopic expression of EGFR significantly attenuated senescence induced by Nutlin-3a (Fig. 7g, h). Correspondingly, tumor reduction by Nutlin-3a treatment could no longer be detected in U87 glioblastoma grafts expressing EGFR (Fig. 7i).

Taken together, these results indicate that the negative regulation of DYRK1A-EGFR axis by p53 also operates in vivo and may suppress tumor growth by promoting cellular senescence.

## Discussion

EGFR signaling is frequently deregulated in cancer and contributes to various features of malignancy. Mutation or loss of p53 is the most common genetic alterations in human malignancy. We here showed that EGFR can be downregulated by p53 in a subset of cancer cell lines and demonstrated downregulation of EGFR as a novel mechanism underlying the cellular senescence caused by p53 activation. Furthermore, we identified p53-MDM2 pathway as a negative regulator of DYRK1A-EGFR axis. MDM2 was shown to catalyze the ubiquitination of DYRK1A. Importantly, we also observed a downregulation of EGFR and an induction of cellular senescence in tumor xenografts in mice treated with Nutlin-3a. These findings provide new insights into the regulation of EGFR in cancer cells and may bear clinical implications.

Our results indicate that the regulation of EGFR by p53 operates distinctly in different cell lines. Increased level of EGFR mRNA level may be accompanied by a reduced protein amount, as in U2OS cells. On the other hand, decreased amount of EGFR transcripts was even associated with increased protein level upon p53 activation, as in A549 cells. As reported in previous studies^27,32^, our results confirmed DYRK1A as a critical stabilizer of EGFR. Unlike the downregulation of DYRK1A by p53 via miR-1246 in A2780 ovarian cancer cells^30^, the negative regulation of DYRK1A by p53 was shown to be mediated by the classical p53 target MDM2, at least in U2OS and U87 cells. The results shown here as well as in previous studies indicate that the mechanisms by which EGFR level is regulated by p53 are complex and diverse.

MDM2 is best known as an antagonist of p53. By ubiquitinating and destabilizing p53, MDM2 functions to prevent prolonged or excessive p53 activation^15^. MDM2 has also been found to exhibit p53-independent functions^13–21^. The substrates ubiquitinated by MDM2 include FOXO3A, DHFR, SNAIL, and H2A, and are subjected to either polyubiquitination or monoubiquitination. Those substrates participate in diverse biological processes, such as cell cycle progression, metabolism, redox homeostasis, epithelial–mesenchymal transition, and epigenetic regulation. We here demonstrated DYRK1A as a novel substrate of MDM2. Tight regulation of DYRK1A is critical for neural development and cognitive function, and DYRK1A overexpressed in individuals with Down’s syndrome due to the extra copy of chromosome 21 is believed to underlie the pathogenesis of neurodevelopmental delay, motor abnormalities, and cognitive deficits^36,37^. Therefore, in addition to helping understand the regulation of EGFR in cancer cells, our finding of MDM2 as a negative regulator of DYRK1A may also bear implications in the management of the conditions associated with DYRK1A overexpression. It should be noted that DYRK1A can phosphorylate p53 and augment its transcriptional activity in embryonic neuronal cells^38^. The downregulation of DYRK1A by p53 mediated by MDM2 may therefore represent a negative feedback loop.

Prolonged p53 activation drives cellular senescence in some cell types^39,40^. Many effectors of cellular senescence are transcriptional targets of p53^41–44^. We previously showed that downregulation of EGFR is sufficient to induce cellular senescence^31^. We here demonstrated that p53 activation can also drive senescence via repressing EGFR, though mediated by MDM2 and DYRK1A en route. It should be noted that this senescence-driving pathway by p53 does not operate in all cell lines examined. Whether or not the DYRK1A-EGFR axis responds to p53 activation is apparently context-dependent. It appears that the diverse mechanisms by which EGFR level is regulated by p53 remains to be fully elucidated.

## Materials and methods

### Cells and cell culture

U2OS, U87 MG, A172, A2780, MCF-7, HT1080, LoVo, A549, and HCT116 human cancer cell lines were obtained from the Cell Bank of Chinese Academy of Sciences (Shanghai). All experiments were performed using cells within 20 passages after receipt. The cells were maintained in RPMI-1640, minimal essential medium, or Dulbecco’s modified Eagle’s medium (Gibco, Invitrogen, Thermo Fisher Scientific, Waltham, MA, USA) supplemented with 10% fetal calf serum, 100 U/mL penicillin, and 100 μg/mL streptomycin in a humidified 5% CO_2_/95% air atmosphere at 37 °C. Human peripheral blood mononuclear cells (PBMCs) were obtained from four young, healthy, nonsmoking volunteers and were collected aseptically in the presence of EDTA. Blood collection was carried out in accordance with the Code of Ethics of the World Medical Association (Declaration of Helsinki). Human PBMCs were isolated from blood samples as per the instructions given in the manual provided with Percoll (Solarbio, Beijing, China). Isolated PBMCs were suspended in RPMI-1640 medium with 10% sterile fetal bovine serum, 1% penicillin–streptomycin solution. PBMCs were adjusted to 1× 10^6^ cells/mL and used for further experiments.

### Chemicals

Nutlin-3a and Erlotinib were purchased from Selleck chemicals (Houston, TX, USA). MG132 was from Beyotime Biotechnology (Shanghai, China). Chloroquine was from Abcam (Cambridge, UK), and all other chemicals were of analytical grade and purchased from Sigma Chemical (Burlington, USA). For in vitro and in vivo studies, Nutlin-3a was reconstituted in dimethyl sulfoxide (DMSO) and 2% DMSO/1% hydroxyethyl cellulose/0.2% Tween-80, respectively.

### Flow cytometry analysis of EGFR protein level

Control and treated cells were harvested and washed once with cold phosphate-buffered saline (PBS). Cells were incubated with EGFR antibody (Cat# 352905, BioLegend) in 100 μL PBS at room temperature for 15 min. Cells were washed two times with PBS, and the EGFR protein level was examined by FACScan flow cytometer (Becton Dickinson, San Jose, CA, USA). In each measurement 10,000 viable cells were analyzed.

### EdU incorporation

EdU (Cell-Light™ EdU Cell Proliferation Detection Kit, Guangzhou RiboBio, China) was added at 50 μM and the cells were cultured for an additional 2 h. After the removal of EdU-containing medium, the cells were fixed with 4% paraformaldehyde at room temperature for 30 min, washed with glycine (2 mg/mL) for 5 min in a shaker, treated with 0.2% Triton X-100 for 10 min, and washed with PBS twice. Click reaction buffer (Tris-HCl, pH 8.5, 100 mM; CuSO_4_, 1 mM; Apollo 550 fluorescent azide, 100 μM; ascorbic acid, 100 mM) was then added. After 10–30 min, the cells were washed with 0.5% Triton X-100 for three times, stained with 4′,6-diamidino-2-phenylindole (DAPI) for 10 min at room temperature, washed with 0.5% Triton X-100 for five times, and, finally, immersed in 150 μL PBS and examined under a fluorescence microscope.

### Senescence-associated acidic β-galactosidase staining

A Senescence β-Galactosidase Staining Kit purchased from Cell Signaling Technology (Beverly, MA, USA) was employed. Four high-power fields per sample were counted in three-independent samples to score the number of senescent cells.

### Western blot analysis

Cells were harvested after treatment, rinsed in ice-cold PBS, and lysed in lysis buffer containing 50 mmol/L HEPES (pH 7.9), 0.4 mol/L NaCl, 1 mmol/L EDTA, 2 mg/mL leupeptin, 2 mg/mL aprotinin, 5 mg/mL benzamidine, 0.5 mmol/L phenylmethylsulfonylfluoride, and 1% NP-40. The lysates were centrifuged at 12,000 rpm to remove any cellular debris. Protein concentrations of the lysates were determined by the BCA protein assay system (Beyotime, China). Equal amounts of protein were separated by 10% sodium dodecyl sulfate-polyacrylamide gel electrophoresis, transferred to PVDF membrane (Millipore, Billerica, MA, USA), and blocked with 5% nonfat dry milk in TBS-Tween-20 (0.1%, v/v) for 1 h at room temperature. The membrane was incubated with primary antibody overnight. Antibodies to DYRK1A, His-tag, p53, MDM2, and p21 were purchased from Santa Cruz Biotechnology (Santa Cruz, CA, USA); to Flag, HA, SPRY2, p-ERK, ERK were from Cell Signaling Technology (Beverly, MA, USA); to EGFR was from Abcam (Cambridge, UK); to glyceraldehyde 3-phosphate dehydrogenase (GAPDH) was from Chemicon (Temecula, CA, USA). After washing, the membrane was incubated with the appropriate horseradish peroxidase secondary antibody (diluted 1:5000; Amersham Pharmacia Biotech, Arlington Heights, IL, USA) for 1 h. Following several washes, the blots were developed by enhanced chemiluminescence (Millipore, Billerica, MA, USA).

### Small interfering RNA

EGFR small interfering RNA (siRNA) duplex 1, 2, and 3 (Cat. No. SASI-Hs01-00215450, SASI-Hs01-00010279, SASI-Hs01-00010280), DYRK1A siRNA duplex 1, 2, and 3 (Cat. No. SASI-Hs01-00123259, SASI-Hs01-00123260, SASI-Hs01-00123261), MDM2 siRNA duplex 1, 2, and 3 (Cat. No. SASI-Hs01-00132038, SASI-Hs01-00132040, SASI-Hs01-00132041), and UNIV NEGATIVE CONTROL siRNA were synthesized (Sigma) and transfected into U2OS cells using Lipofectamine 2000 (Invitrogen) for 48 h.

### Plasmids and lentiviral transduction

Vector for overexpression (EX-H3199-M77) of DYRK1A was purchased from GeneCopoeia Inc. (GeneCopoeia, Rockville, MD, USA). Expression vector for p53 (TP53) was purchased from OriGene (Beijing, China). Expression vectors for wild-type (pCMV-myc3-HDM2) and mutant MDM2 (pCMV HDM2(C464A)) were purchased from Addgene (Cambridge, MA, USA). pBabe-EGFR vector from Addgene was used as a PCR template for cloning of EGFR, and PCR products were cloned into *Not*I/*Mlu*I sites of lentiviral vector (pLVX-TRE 3G). The EGFR insert was verified by sequencing. Lentiviral particle production and infection were performed as described previously^10^. Briefly, lentiviral vectors were cotransfected with psPAX2 and pMD2G vectors into HEK293T cells. Supernatants were collected at 24 and 48 h after transfection and stored in −80 °C. For transduction, 3 ×10^4^ cells per well were seeded in six-well plates and infected with lentiviruses the following day. The medium was removed 24 h later and replaced with fresh medium containing 1 μg/mL puromycin. Cells were selected for about 2 weeks to generate stable cell lines. Lentivirus shp53 (shp53-pLKO.1-puro, Addgene) were prepared in HEK293T cells packaged by pMD2G and psPAX2. For stable infection, 3 × 10^4^ cells were plated in each well of the six-well plates along with 2 mL of medium without antibiotics. After overnight incubation, the medium was removed and replaced with 1 mL per well of medium containing lentivirus After 24h, fresh medium containing 2 μg/mL puromycin was added to each well for stable infection. Cells were selected for about 2 weeks to generate stable cell lines.

### cDNA synthesis and real-time PCR

mRNAs or microRNAs (miRNAs) were reverse transcribed to generate complementary DNA (cDNA) using oligo(dT) primers or stem-loop reverse transcriptase (RT) primers, respectively. Then, U6 small nuclear RNA (for miRNA) or GAPDH (for mRNA) was considered as the endogenous control. microRNAs were isolated from total RNA using the All-in-One™ miRNA qRT-PCR Detection Kit (GeneCopoeia, Rockville, MDd, USA), according to the manufacturer’s instructions. Quantitative reverse transcriptase-polymerase chain reaction was performed using LightCycler Fast Start DNA Master SYBR Green I on a LightCycler (Roche Diagnostics GmbH, Mannheim, Germany) system. The primers used in the present study were as follows: EGFR, 5′-AGGCACGAGTAACAAGCTCAC-3′ and 5′-ATGAGGACATAACCAGCCACC-3′; p21, 5′-CGATGGAACTTCGACTTTGTCA-3′ and 5′-GCACAAGGGTACAAGACAGTG-3′; GAPDH, 5′-CAGAACATCATCCCTGCCTCTAC-3′ and 5′-TTGAAGTCAGAGGAGACCACCTG-3′.

### Luciferase reporter assay

The sequence of promoter and 5′ end of EGFR (from −646 to −91   bp of the transcriptions start site numbered according to Entrez Gene ID 1956, GI: 399923581) was cloned into pGL3-basic vector. For transfection, 2  × 10^4^ U2OS and U87 cells were seeded in duplicate into 96-well culture plates and transfected with 1 μg of reporter construct using Lipofectamine 2000 (Invitrogen) as per the manufacturer’s instructions. Cells were incubated with/without Nutlin-3a (8 μM) for 48 h and then harvested. Firefly and Renilla luciferase activities were measured by using the Dual-Luciferase Reporter Assay System Kit (Promega, Madison, WI, USA) and assessed with a luminometer. Renilla luciferase activity from a cotransfected pRL-TK control vector (50 ng/well) was used for normalization.

### Immunoprecipitation ubiquitination analysis

Cells were collected with lysis buffer ((50 mM Tris (pH 8.0), 150 mM NaCl, 1 mM EDTA, 1% NP-40, 1 mM dithiothreitol, 1 mM phenylmethylsulfonyl fluoride, 1 mM Na_3_VO_4_, and 1 mg/mL each of leupeptin, pepstatin, and aprotinin). One milligram of proteins was then immunoprecipitated overnight at 4 °C with the indicated antibody or Ni-NTA His Bind Resin (Sangon Biotech, Shanghai, China). After extensive washing with lysis buffer, the immunocomplexes were analyzed by western blotting assay. For ubiquitination assays, HEK293T cells or U2OS cells were cotransfected with plasmids encoding HA-Ub, His-tagged DYRK1A, and the indicated TP53, Myc3-MDM2 WT or MDM2 C464A, siRNA duplexes specific to MDM2 or negative oligo. Followed by treated with MG132, the cell lysates were prepared, and DYRK1A or p53 precipitates were isolated with Ni-NTA or protein A/G-agarose, and then subjected to western blotting.

### In vivo tumor xenografts

For generation of orthotopic U87 xenografts, cells were collected from cultures at 80% confluence, resuspended in PBS containing 0.05% trypsin and 0.02% EDTA, and centrifuged at 600 × *g* for 5 min at room temperature. Cell pellets were then resuspended in fresh culture medium and rapidly counted in a Burker chamber. After a second centrifugation in the same conditions, the cells were resuspended in serum-free medium. 5- to 6-week-old BALB/c nude mice (Vital River Laboratories, Beijing, China) mice were anesthetized with phenobarbital sodium (60 mg/kg, i.p.). Animals received approximately 1.5× 10^5^ U87 cells (>95% viability) in a volume of 3 μL stereotactically injected in the right caudate nucleus: bregma (anatomical point on the mouse skull at which the coronal suture is intersected perpendicularly by the sagittal suture), 0.5 mm; lateral, 1.75 mm. The needle was initially advanced to a depth of 3.5 mm and then withdrawn to a depth of 3 mm to limit reflux up the needle tract during injection of cells. Drug treatment was begun 5 days after tumor inoculation. Mice were dosed by i.p. injection with the vehicle alone (con) or Nutlin-3a (40 mg/kg body weight) every other day (*n* = 5–6 per group). Brains were fixed with 4% paraformaldehyde in PBS at 4 ℃ overnight, dehydrated in 20% sucrose until the tissue sinks, embedded in OCT (Fisher), and then sectioned for hematoxylin and eosin (H&E) staining, immunofluorescence, and senescence assay. The following formula was used for tumor volume measurement: tumor volume = *L* × *W*^2^/2, where *L* is the length and *W* is the width. All animal procedures were approved by the Institutional Animal Care and Use Committee of Shandong University.

### Immunofluorescence analysis

For cells, cells were grown on coverslips and were washed in PBS twice and fixed in 4% paraformaldehyde for 20 min at room temperature, and then permeabilized in 0.2% Triton X-100 for 10 min. For frozen sections (7 µm), slips were washed with PBS twice. Cells or frozen sections were blocked with 10% goat serum in PBS at 37 °C for 1 h, following which rabbit anti-EGFR antibody (Abcam), rabbit anti-DYRK1A antibody (Santa Cruz), mouse anti-p21 antibody (Santa Cruz), and rabbit anti-Lamin B1 antibody (Proteintech) were added at a dilution of 1:200 in 5% bovine serum albumin in PBS, respectively, and incubated overnight at 4 °C. Cells or sections were then washed thrice in PBS before incubating in the dark with secondary antibody at a dilution of 1:300 in 5% bovine serum albumin in PBS for 60 min. The secondary antibody solution was then aspirated and the sections were washed four times in PBS. Cells or sections then were incubated in the dark with 4′, 6-diamidino-2-phenylindole (1 mg/mL) in PBS for 5 min and coverslips were mounted with an antifade solution. Slides were then examined on a Leica fluorescence microscope. Images were captured by a charge-coupled device camera. Negative controls were performed by omitting the primary antibodies.

### Statistical analysis

Results were expressed as mean ± SD. Statistical calculations were performed using the SigmaPlot 2000 software (Systat Software, San Jose, CA, USA). Differences in measured variables between experimental and control groups were assessed using *t* test. *P* < 0.05 was considered statistically significant, and levels of significance were presented at *P < 0.05, **P < 0.01, and ***P < 0.001, respectively.

## Supplementary information

Legends to supplemental figures

Figure S1

Figure S2

Figure S3

Figure S4

Figure S5

Figure S6

Figure S7

Figure S8

Figure S9

Figure S10
