# Supplementary material for: Inhibition of DYRK1A-EGFR axis by p53-MDM2 cascade mediates the induction of cellular senescence
Source: Cell Death Dis. 2019 Mar 25;10(4):282. doi: 10.1038/s41419-019-1521-5 (PMC6433862; doi:10.1038/s41419-019-1521-5)
Supplement: Supplementary file 1 — Legends to supplemental figures [file 41419_2019_1521_MOESM1_ESM.docx]

**Fig.S1.** Differential regulation of EGFR by Nut3a in A549, MCF-7, LoVo and HCT116 cells. (A) A549, (B) MCF-7, (C) LoVo, and (D) HCT116 cells. Cells were treated with Nut3a for the indicated time and dose, and then equal amounts of cell lysates were subjected to immunoblot analysis using the indicated antibodies. The immunoblots are representative of at least two independent experiments with GAPDH serving as a protein loading control.

**Fig.S2.** EGFR was downregulated by Doxorubicin(Doxo), Cisplatine(Cis) and X-ray . (A)The protein levels of EGFR, DYRK1A and p53 were measured in U2OS cells treated with Doxo(0.2μM) or Cis(5μM) for 3 d. (B) EGFR was measured by western blot in U87 cells exposured with 10 Gy of X-ray for the indicated days. (C) EGFR is unaffected by p53 activation in normal human fibroblasts. (D) The protein levels of EGFR from monocytes were determined by ﬂow cytometry (8μM Nut3a for 72 h). U87 cells as a positive control. Right, quantitative summary of EGFR expression by flow cytometry was shown. (E) The protein levels of EGFR from lymphocytes (without PHA) were determined by ﬂow cytometry (8μM Nut3a for 72 h). U87 cells as a positive control. Right, quantitative summary of EGFR expression by flow cytometry was shown. (F) Lymphocytes were stimulated with PHA and the protein levels of EGFR were determined by ﬂow cytometry (8μM Nut3a for 72 h). U87 cells as a positive control. Right, quantitative summary of EGFR expression by flow cytometry was shown. Representative results of three independent experiments were shown.

**Fig.S3.** Transcriptional activation or repression of EGFR by p53 in different cell lines.

(A, B) Left, qRT-PCR measurements of p21 and EGFR transcrits in U87 and U2OS cells treated with the indicated [concentration](javascript:void(0);) of Nut3a for 72 h. Right, measurements of the promoter activity of EGFR in U87 and U2OS cells. Cells were co-transfected with either pGL3-EGFR promoter or pGL3 basic vector, and Renilla luciferase reporters. Transfected cells were incubated with the indicated concentration of Nut3a for 48 h. (C, D, E) qRT-PCR measurements of p21 and EGFR transcripts in A2780, A172 and HT1080 cells treated with the indicated [concentration](javascript:void(0);) of Nut3a for 72 h. Data are the Mean±SD from quadruplicates. Three independent experiments yielded reproducible results.*, *p* < 0.05 versus control and **, *p* < 0.01 versus control.

**Fig.S4.** The mRNA level of EGFR was decreased in Nut3a-treated A549 cells. The mRNA levels of p21 and EGFR were measured by qRT-PCR after cells were treated with indicated [concentration](javascript:void(0);)s of Nut3a for 72 h in A549 cells.

**Fig.S5.** p53 activation inhibits cell proliferation. (A) Proliferation was determined by EdU incorporation assay after cells were exposed to Nut3a for 7 d in U87 cells. Right, quantitative summary of EdU incorporation was shown. (B) Quantitative summary of EdU incorporation in U2OS cells. At least 200 nucleated cells were scored. (C) The protein levels of p16 were determined by western blot.U87 and U2OS cells were treated with the indicated time of 8μM Nut3a.

**Fig.S6.** EGFR downregulation-dependent and -independent induction of cellular senescence by p53 activation in different cell lines. (A, B) EGFR siRNA or EGFR inhibitor induced senescence in HT1080 and A172 cells. Top, depletion of EGFR by siRNA was measured by western blot. Bottom, quantitative summary of senescent cells. Cellular senescence was examined 7 d after RNAi of EGFR or EGFR inhibitor (Erlotinib, 10μM). (C, D) Left, Nut3a induced cellular senescence in A549 and MCF-7 cells. Right, inhibitory effect of Nut3a on cell proliferation was determined by EdU incorporation assay (7 d after Nut3a exposure). Data are presented as averages of triplicate measurements.

**Fig.S7.** DYRK1A downregulation by Nut3a is not mediated by miR-1246. (A) qRT-PCR assay of DYRK1A mRNA was measured in U2OS, U87, A2780 and HT1080 cells. (B) qRT-PCR assay of miR-1246 and western blot analysis of DYRK1A expression in U2OS cells transfected with the miRNA mimic. (C) Left, qRT-PCR assay of miR-1246 in U2OS cells treated with the indicated concentration of Nut3a, A2780 cells as a positive control. Right, qRT-PCR measurement of miR-1246 in U2OS cells treated with Nut3a for the indicated durations.

**Fig.S8.** Downregulation of EGFR by p53 activation is attenuated by chloroquine.

(A) U2OS, (B) HT1080, (C) U87 cells. Cells were treated with 8μM Nut3a for 48 h, and were then treated with 25μM chloroquine for the last 6 h before harvest. Protein levels of p53, DYRK1A, EGFR were analyzed by western blot. (D) Ectopic expression of MDM2 downregulates DYRK1A. pCMV-myc3-HDM2 expression vector were transfected into HEK293T cells for 48 h, cells extracts were examined by western blot for the determination of Myc, MDM2, p53 and DYRK1A.(E) DYRK1A is downregulated in U87 and A172 cells where MDM2 upregulation after p53 activation. MDM2, p53 and DYRK1a were measured by western blot in U87 and A172 cells exposured with 8μM Nut3a for the indicated days.

**Fig.S9.** MDM2 catalyzes p53 ubiquitination. HEK293T cells were transfected with HA-Ub and TP53 in the presence of MG132 for 6 h. and cell lysates were then immunoprecipitated with p53 antibody followed by immunoblotting with an anti-ubiquitin antibody.

**Fig.S10.** Nut3a downregulates EGFR and DYRK1A in glioblastoma in vivo. U87 cells were inoculated into the right striatum of mouse brain (n=5-6 per group). (A) Representative IF images showing the expression of p21 after treatment of Nut3a (×400). Scale bar, 10μm. Right, quantitative immunointensity of p21 was analyzed by ImageJ software (NIH, Bethesda, MD). (B) Representative IF images showing the expression of DYRK1A (×400). Right, quantitative immunointensity of DYRK1A were analyzed by ImageJ software. (C) Left, Representative IF images showing the expression of EGFR (×400). Scale bar, 10μm. Right, quantitative immunointensity of EGFR was analyzed by ImageJ software. **, *p* < 0.01, ***, *p* < 0.001 versus control.
