# Supplementary figures and images for: Inhibition of DYRK1A-EGFR axis by p53-MDM2 cascade mediates the induction of cellular senescence
Source: Cell Death Dis. 2019 Mar 25;10(4):282. doi: 10.1038/s41419-019-1521-5 (PMC6433862; doi:10.1038/s41419-019-1521-5)

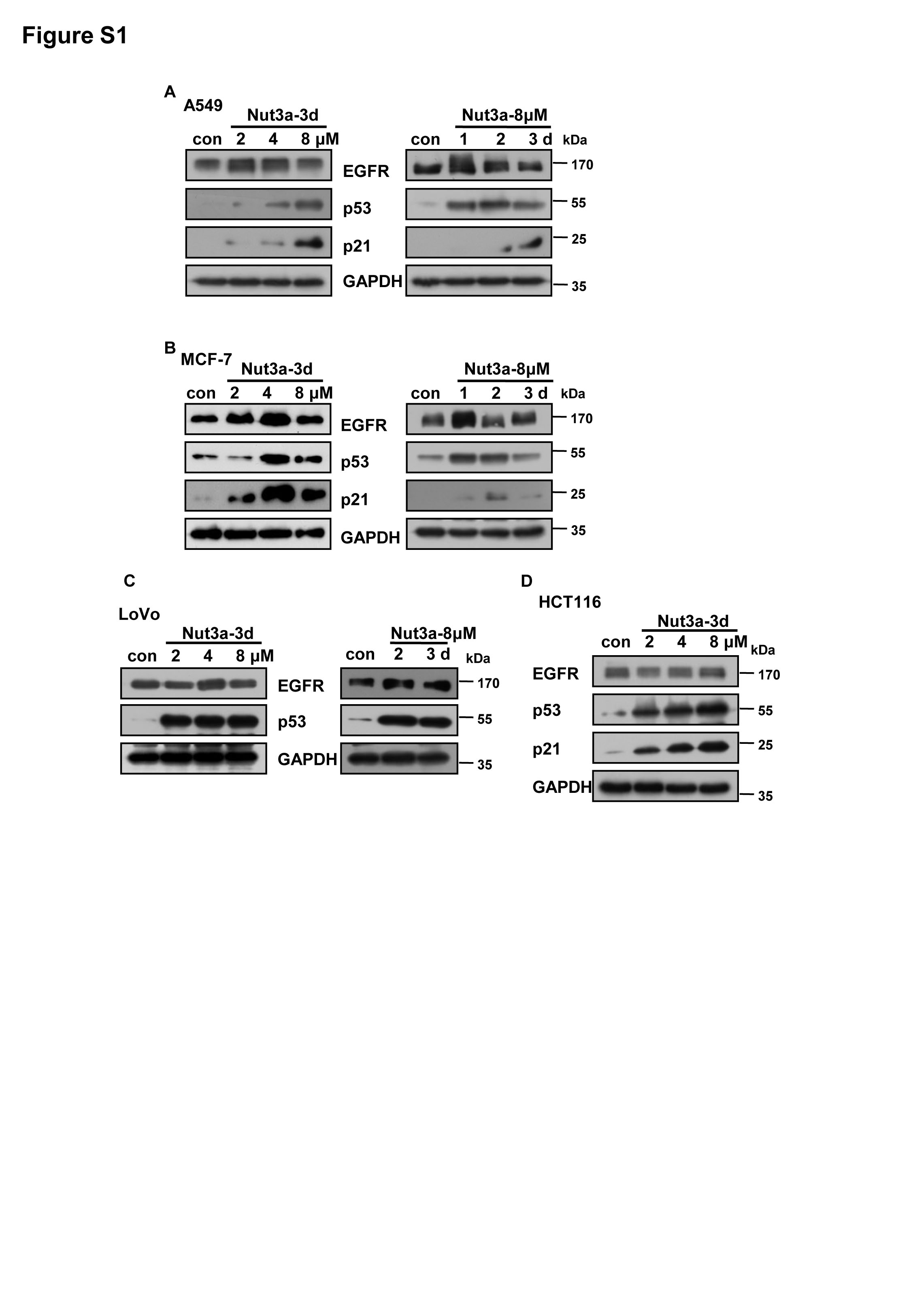

Supplement: Supplementary file 2 — Figure S1 [file 41419_2019_1521_MOESM2_ESM.jpg]

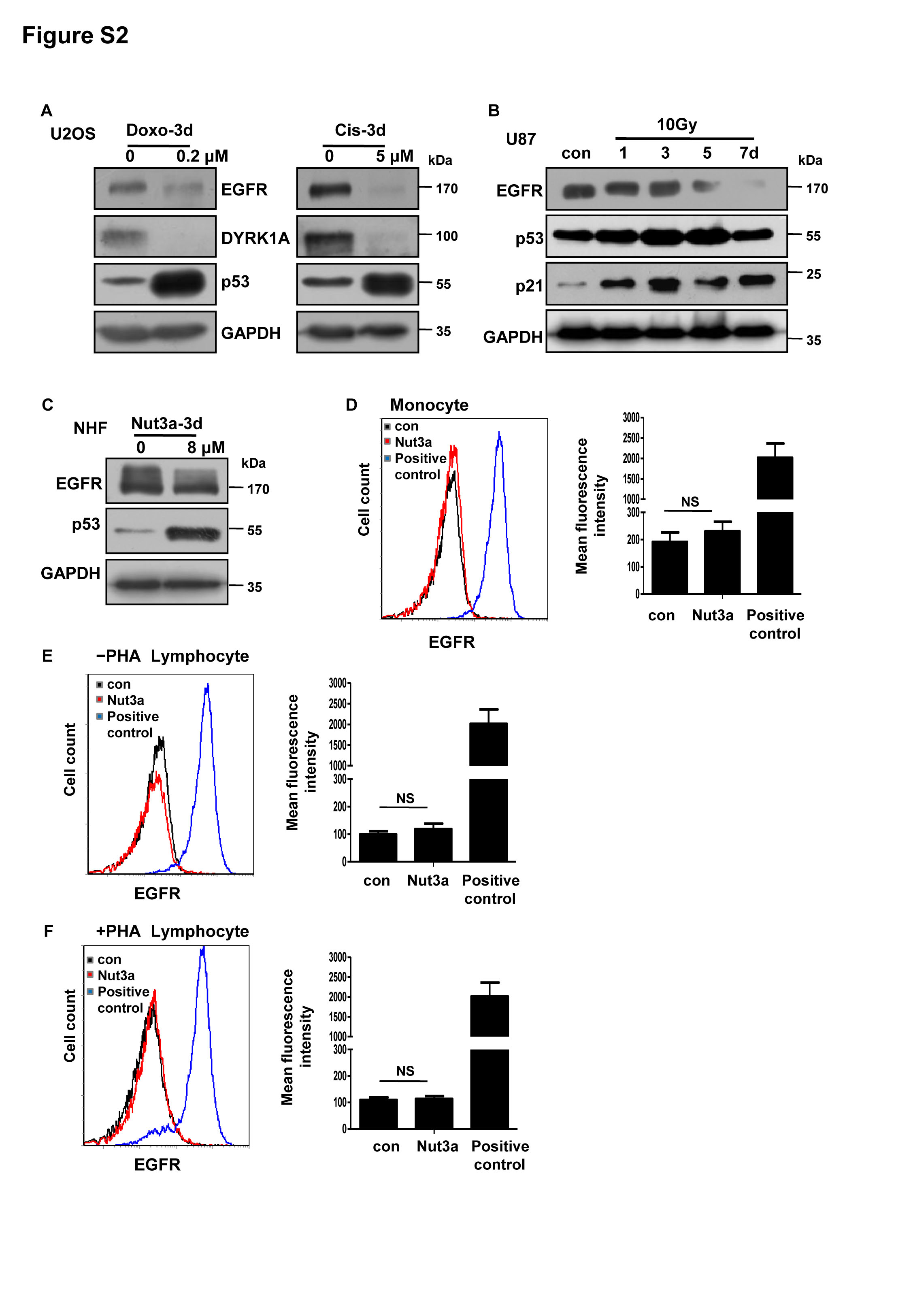

Supplement: Supplementary file 3 — Figure S2 [file 41419_2019_1521_MOESM3_ESM.jpg]

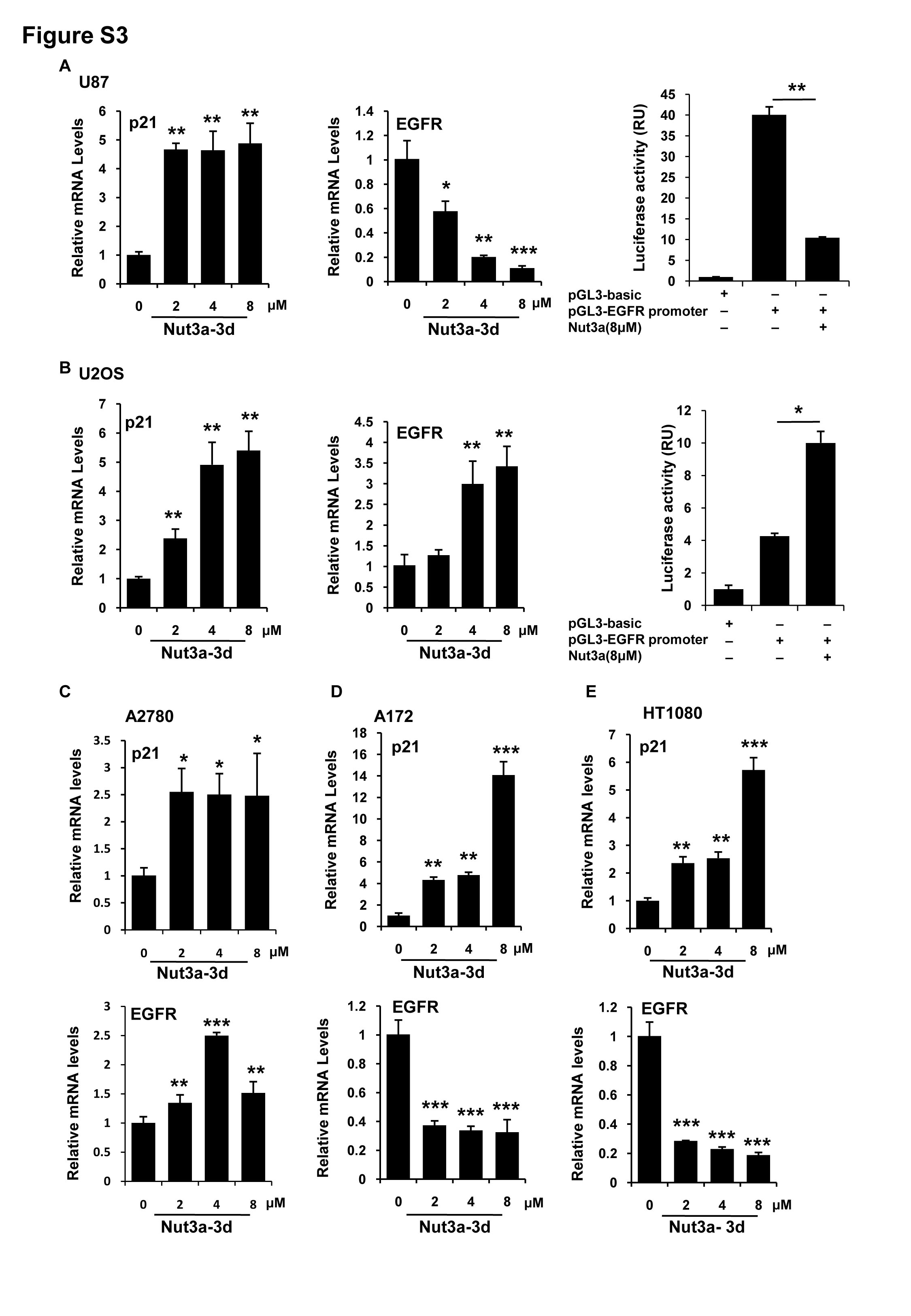

Supplement: Supplementary file 4 — Figure S3 [file 41419_2019_1521_MOESM4_ESM.jpg]

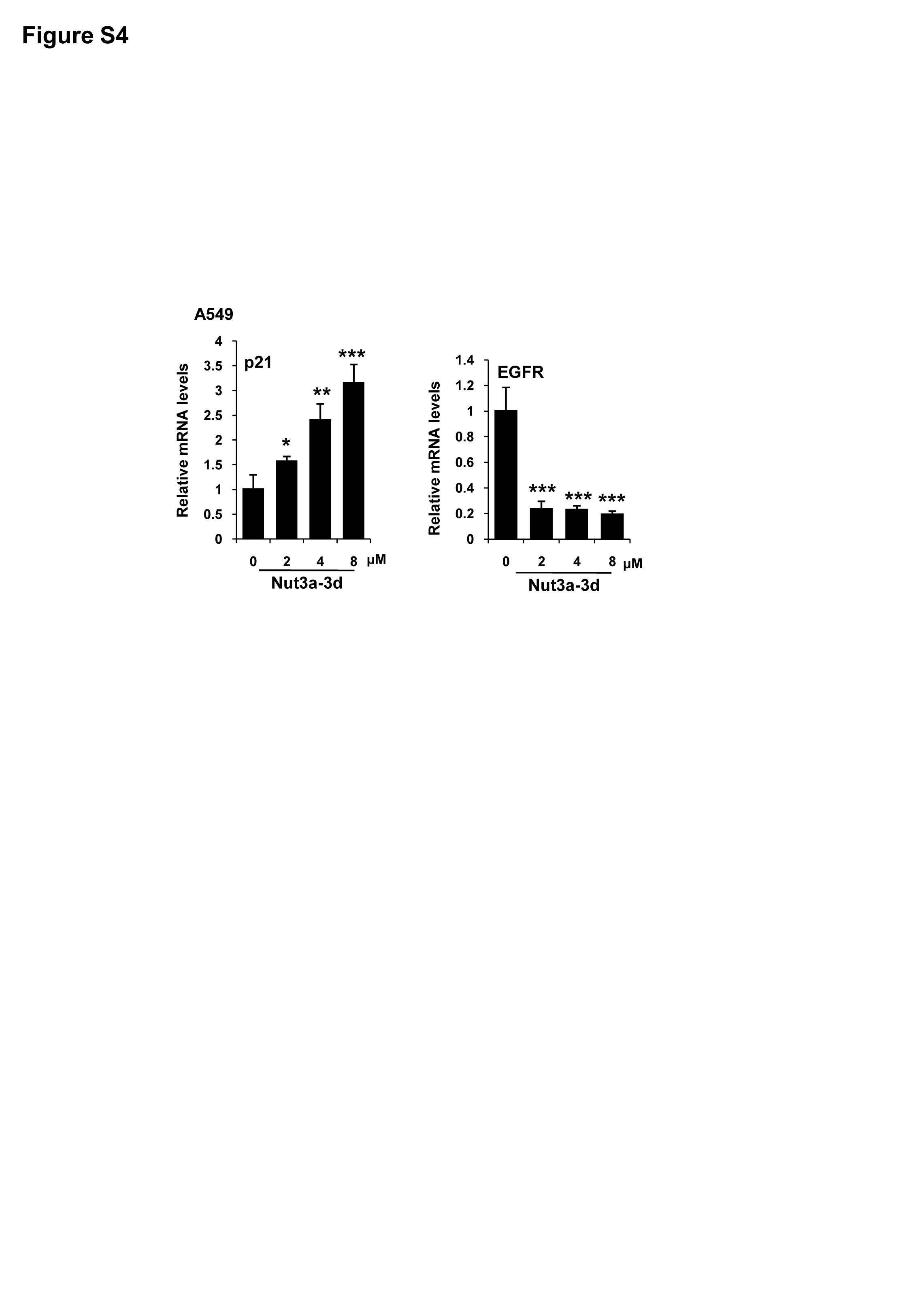

Supplement: Supplementary file 5 — Figure S4 [file 41419_2019_1521_MOESM5_ESM.jpg]

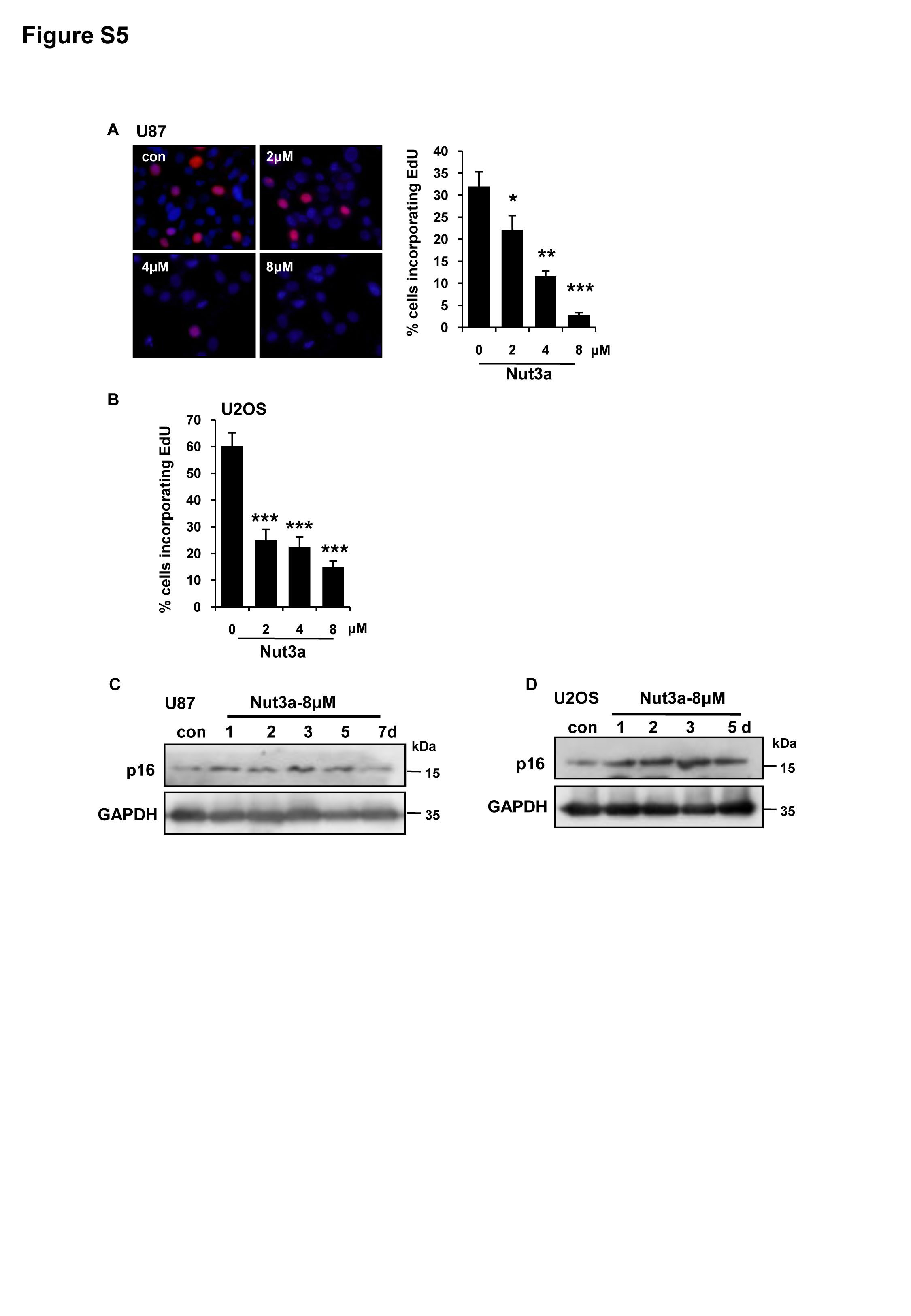

Supplement: Supplementary file 6 — Figure S5 [file 41419_2019_1521_MOESM6_ESM.jpg]

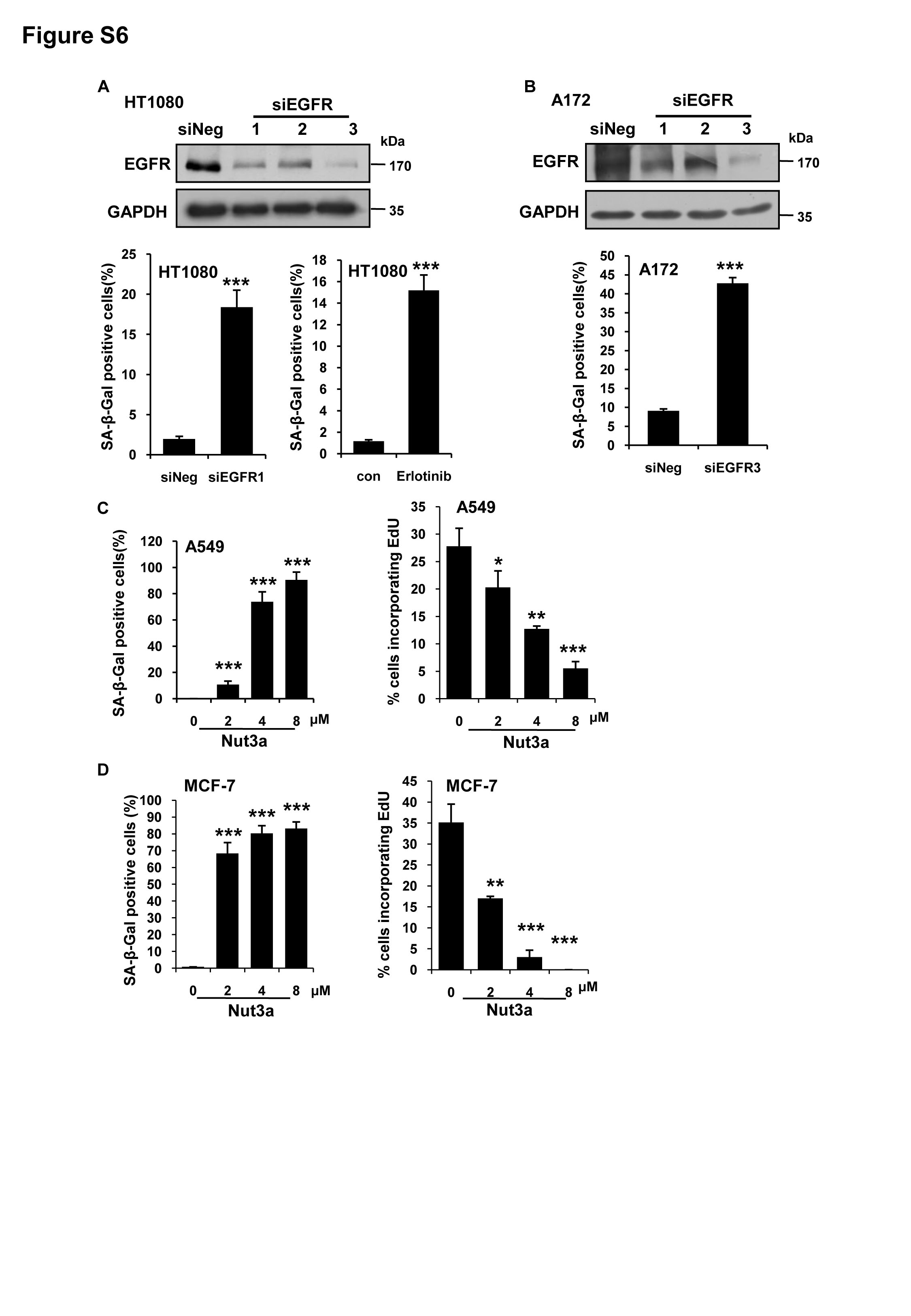

Supplement: Supplementary file 7 — Figure S6 [file 41419_2019_1521_MOESM7_ESM.jpg]

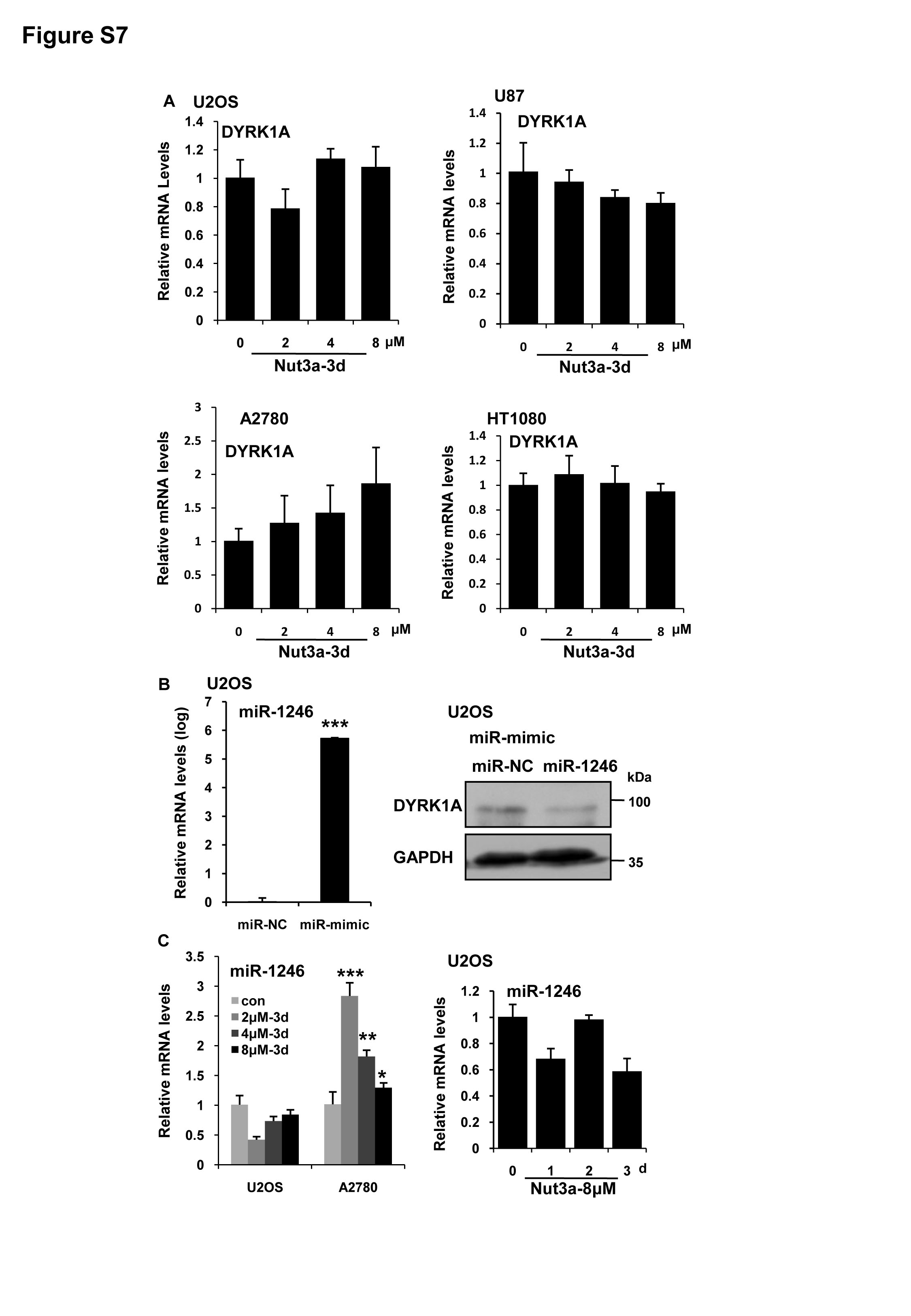

Supplement: Supplementary file 8 — Figure S7 [file 41419_2019_1521_MOESM8_ESM.jpg]

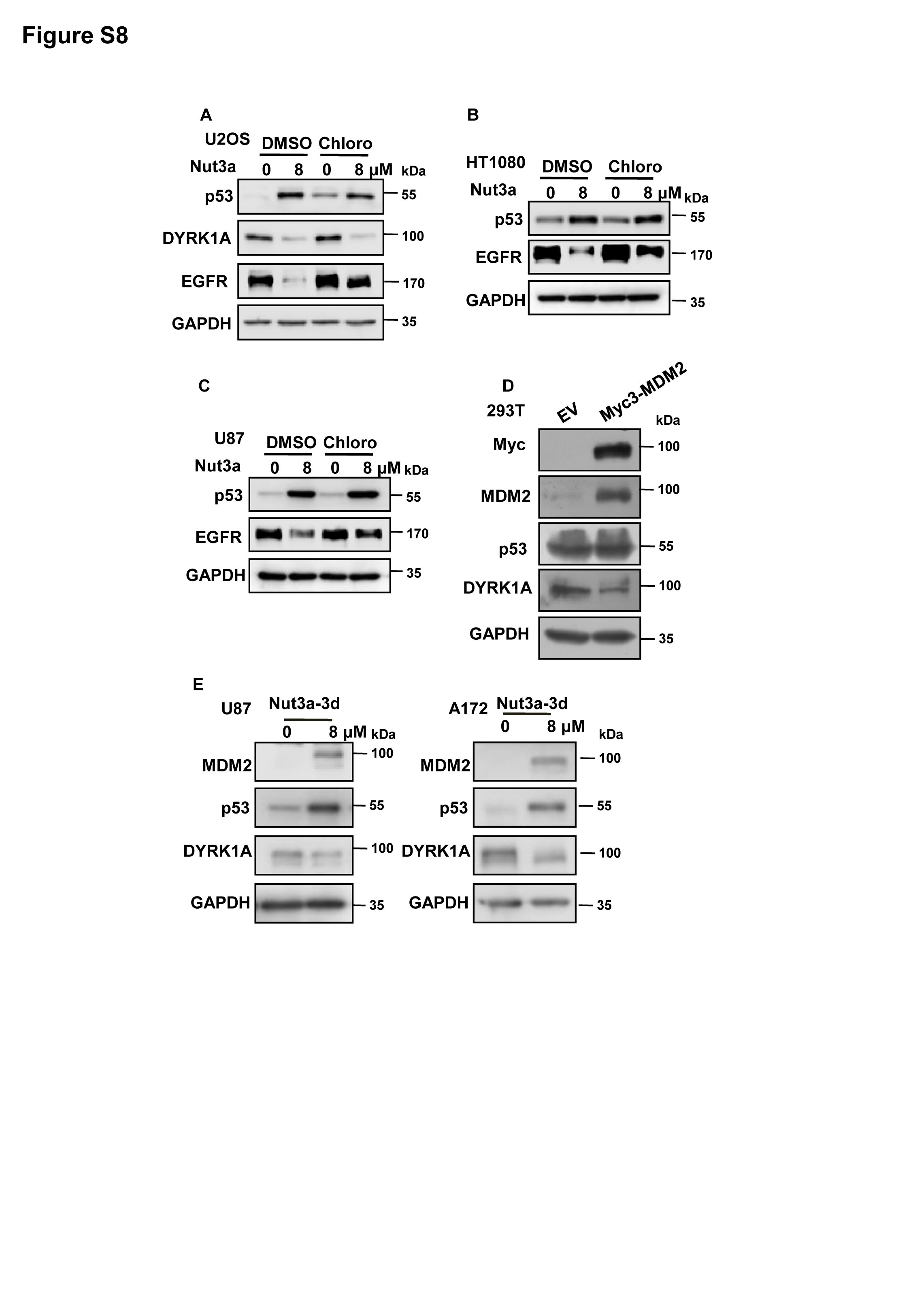

Supplement: Supplementary file 9 — Figure S8 [file 41419_2019_1521_MOESM9_ESM.jpg]

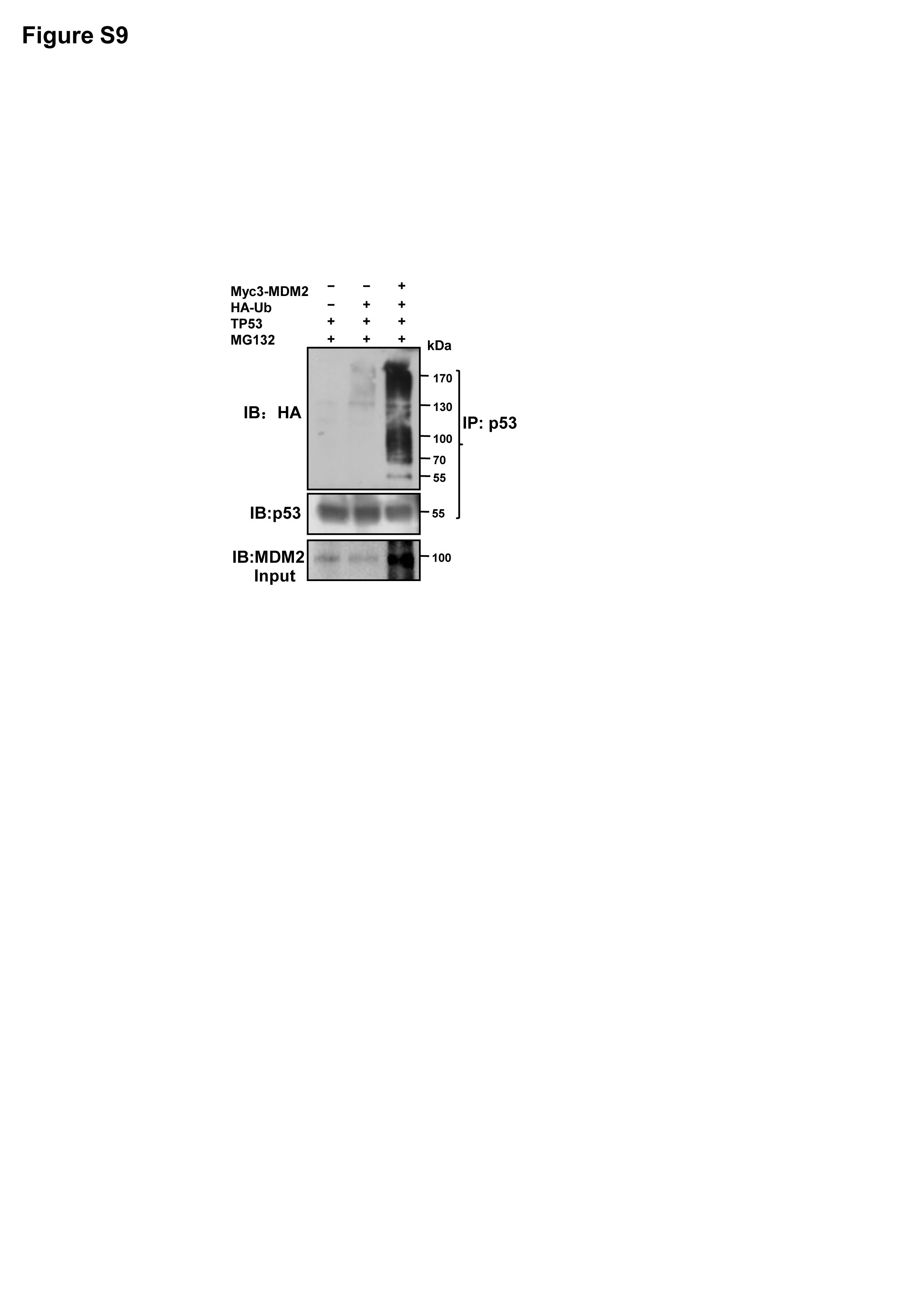

Supplement: Supplementary file 10 — Figure S9 [file 41419_2019_1521_MOESM10_ESM.jpg]

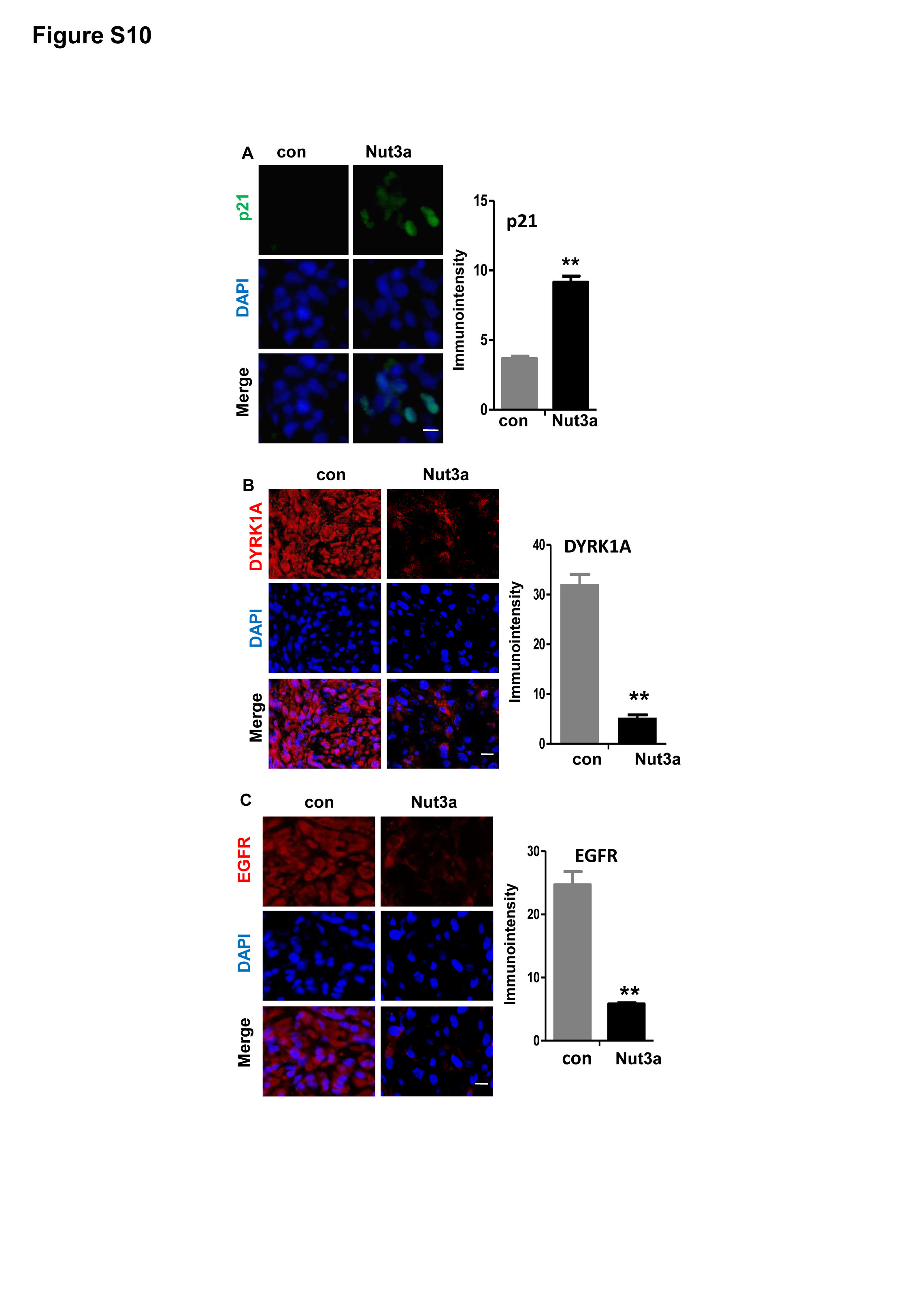

Supplement: Supplementary file 11 — Figure S10 [file 41419_2019_1521_MOESM11_ESM.jpg]
